# Functional Genomic Insights into Probiotic *Bacillus siamensis* Strain B28 from Traditional Korean Fermented *Kimchi*

**DOI:** 10.3390/foods10081906

**Published:** 2021-08-17

**Authors:** Sojeong Heo, Jong-Hoon Kim, Mi-Sun Kwak, Do-Won Jeong, Moon-Hee Sung

**Affiliations:** 1 Department of Food and Nutrition, Dongduk Women’s University, Seoul 02748, Korea; hsjeong32@hanmail.net; 2 Department of Bio and Fermentation Convergence Technology, Kookmin University, Seoul 02707, Korea; jh9261@naver.com (J.-H.K.); mskwak@kookmin.ac.kr (M.-S.K.); 3 KookminBio Corporation, Seoul 02826, Korea

**Keywords:** *Bacillus siamensis*, strain B28, kimchi, probiotic, genome

## Abstract

*Bacillus siamensis* strain B28 was previously isolated from traditional Korean fermented *kimchi* and inhibited expression of the microphthalmia-associated transcription factor and β-catenin in human embryonic kidney 293 cells. Here, we determined the complete genome sequence of strain B28 and compared it with other strains to elucidate its potential probiotic properties. Strain B28 does not contain antibiotic resistance-, hemolysin- or enterotoxin-encoding genes. The genome includes genes related to survival in extreme conditions, adhesion in the gut, and synthesis of the bacteriocin. Considering the potential for enhancement of human health, the strain B28 genome encodes genes related to production of eight essential amino acids, γ-aminobutyric acid, branched-chain fatty acids, γ-glutamyltransferase, and subtilisin. There are genes for the synthesis of uracil, lipoteichoic acid, glutathione, and several reactive oxygen species-scavenging enzymes. Experimentally, strain B28 exhibited sensitivity to eight antibiotics and antibacterial activity against seven foodborne pathogens. *B. siamensis* B28 is a safe strain with potential for development as a probiotic.

## 1. Introduction

The *Bacillus* genus comprises spore-forming bacteria found in various niches, such as oil, agricultural crops, fermented foods, and the human gastrointestinal tract [1,2,3,4,5,6]. *Bacillus* sp. can also be detected in kimchi using culture-dependent and -independent methods [7,8]. *Bacillus* spp. play important roles in the enhancement of the sensory properties of fermented foods via their amylolytic, lipolytic, and proteolytic activities [9,10,11,12,13]. *Bacillus* spp. have been used in the preparation of diverse fermented soybean products in northeast Asia for many centuries [14]. Furthermore, some *Bacillus* strains exhibit antibacterial activity against food pathogenic bacteria [15,16], anticancer effects on human colon cancer cells [17], and probiotic properties in humans and animals [18].

A *B. polyfermenticus* strain named B28 isolated from *kimchi* inhibited the expression of microphthalmia-associated transcription factor and β-catenin by human embryonic kidney 293 cells [19]. However, *B. polyfermenticus* has not yet been placed in the taxonomy database of the National Center for Biotechnology Information at June 2021 (NCBI; http://ncbi.nlm.nih.gov/Taxonomy) or in the List of Prokaryotic Names with Standing in Nomenclature [20]. Recently, several *B. polyfermenticus* strains have been reclassified: *B. polyfermenticus* GF423 was renamed *B. velezensis* variant *polyfermenticus* GF423 based on genome sequencing [21], and *B. polyfermenticus* KMU01 was reclassified as *B. velezensis* KMU01 [15]. Therefore, in this study, we examined the classification of strain B28 based on its whole genome sequence. We also undertook comparative genomic analysis to understand the functionality and safety of strain B28 and to assess its probiotic properties.

## 2. Materials and Methods

### 2.1. Bacterial Strains and Culture Conditions

Strain B28 was grown in tryptic soy broth (TSB; Difco, Detroit, MI, USA) to maintain its traits [15].

### 2.2. Genomic DNA Preparation and Genome Sequencing

Genomic DNA was prepared using a Wizard Genomic DNA Purification Kit (Promega, Madison, WI, USA). Complete genome sequencing was performed using a combination of the Illumina MiSeq system (Illumina, San Diego, CA, USA) and the Single-Molecule Real-Time (SMRT) sequencing system (20 kbp; PacBio, Menlo Park, CA, USA) at ChunLab (Seoul, Korea). The generated SMRT sequencing reads (106,141 reads, 343.12-fold coverage) and MiSeq sequencing reads (6,396,282 reads, 401.906-fold depth) were assembled into three contigs by the MaSurca algorithm (version 3.3.9) [22] and plasmidSPAdes (version 3.14.1) [23]. Gene prediction was performed by Glimmer 3 and NCBI Prokaryotic Genome Annotation Pipeline (version 4.6) [24,25], and functional analysis of the annotated genes was performed using the Clusters of Orthologous Groups (COG) database [26] and SEED database (https://rast.nmpdr.org/rast.cgi accessed on June 2021).

### 2.3. Comparative Genomics

Genome information for *B. siamensis* SCSIO 05746 (GenBank accession no. GCA_002850535.1), *B. siamensis* KCTC 13613^T^ (GenBank accession no. GCA_000262045.1), *B. amyloliquefaciens* FS1092 (GCA_004421045.1), *B. amyloliquefaciens* RD7-7 (GCA_001705195.1), *B. velezensis* JJ-D34 (GCA_000987825.1), and *B. velezensis* KMU01 (GCA_015277495.1) was retrieved from the NCBI database for comparative genomic analysis. The similarity of the core genome was tested via average nucleotide identity (ANI) [27]. To find orthologous genes, the EDGAR (Efficient Database framework for comparative Genome Analyses using BLASTP score Ratios) platform (EDGAR 3.0) was used [28]. Predictions of amino acid metabolic pathways were performed using the RAST (Rapid Annotation using Subsystem Technology) server [29] and iPath (Interactive Pathways Explorer; version 3.0) software [30].

### 2.4. Multilocus Sequence Typing

Multilocus sequence typing (MLST) developed for *Bacillus* was used to identify strain B28 at the species level [31]. The internal regions of eight housekeeping genes, *adk, ccpA*, *glpF*, *gmk*, *ilvD*, *pur*, *spo0A*, and *tpi*, were combined in the order presented; then phylogenetic trees were constructed using MEGA 7 software based on the maximum likelihood method with 1000 bootstrapping values [32]. 

### 2.5. Disc Diffusion Analysis

Antibiotic resistance was determined by the agar disk-diffusion method [33]. In brief, strain B28 was spread onto Muller-Hinton agar with eight antibiotic disks containing clindamycin (10 μg), gentamicin (10 μg), erythromycin (15 μg), lincomycin (15 μg), chloramphenicol (30 μg), tetracycline (30 μg), vancomycin (30 μg), and streptomycin (300 μg) [15]. The plate was incubated at 30 °C for 24 h before being checked for halo formation. 

### 2.6. Hemolytic Activity Tests

β-Hemolytic activity was determined by halo formation around colonies on tryptic soy agar (Difco) supplemented with 5% (*v/v*) sheep blood (MBcell, Seoul, Korea). Clinically isolated *Staphylococcus aureus* USA300-P23 was used as a positive control [34]. The experiments were performed in triplicate.

### 2.7. Enterotoxin Gene Amplification

Seven enterotoxin genes were amplified from genomic DNA using specific primer sets [35]. The PCR reactions were performed using Inclone *Taq* polymerase (Inclone Biotech, Daejeon, Korea) according to the manufacturer’s recommended methods. The amplicons were checked on 1.2% agarose gel. *Bacillus cereus* KCCM 11341 was used as a positive control. 

### 2.8. Determination of Antibacterial Activity

To check the antibacterial activity of strain B28, eight pathogenic bacteria, *B. cereus* KCCM 11341, *Listeria monocytogenes* ATCC 19111, *S. aureus* ATCC 12692, *Alcaligenes xylosoxidans* KCCM 40240, *Flavobacterium* sp. KCCM 11374, *Escherichia coli* O157:H7 EDL 933, *Vibrio parahemolyticus* KCTC 2729, and *Salmonella enterica* KCCM 11862, were used as indicator strains. Pathogens were incubated to an OD_600 nm_ of 1.0, then spread onto TSA with a sterilized paper disk. Strain B28 was cultured in TSB to an OD_600 nm_ of 1.0, and the supernatant was obtained after centrifugation at 5000× *g* for 5 min at 4 °C. After filtration of the supernatant using a 0.22-μm filter, a 15 μL aliquot was dropped onto the disk. The antibacterial activity was determined by the formation of a clear halo around the disk.

### 2.9. Database Accession Numbers

The complete genome sequence of *B. siamensis* B28 was deposited in the DDBJ/ENA/GenBank with accession numbers CP066219–CP066221. The strain was deposited in the Korean Collection for Type Cultures with accession number KCTC 13179BP.

## 3. Results and Discussion

### 3.1. Species Classification of Strain B28

**Figure 1:**
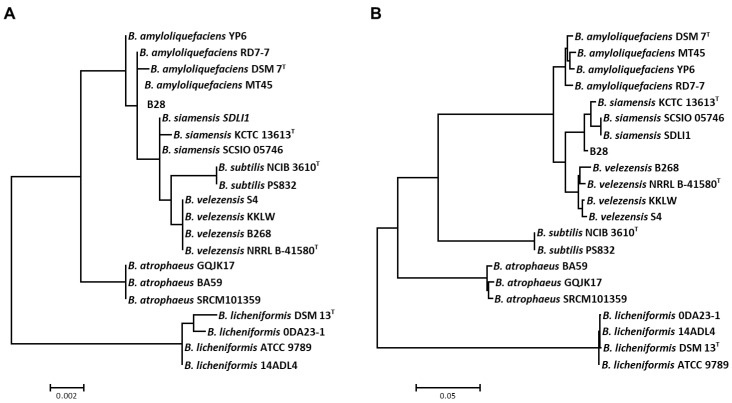
Phylogenetic analysis of 16S rRNA genes (**A**) and multilocus sequence typing (**B**) of strain B28 based on the maximum likelihood method with 1000 bootstrap values. Branches with bootstrap values lower than 50% have been collapsed. The scale of the diagram is the pairwise distance expressed as the percentage dissimilarity.

Strain B28 was isolated from *kimchi* and identified as *B. polyfermenticus* in a previous investigation [19]. However, currently, the 16S rRNA gene sequence of strain B28 had 99.9% identity with those of *B. amyloliquefaciens* MT45 and *B. siamensis* SCSIO 05746 (Figure 1A). By MLST, which is used for discrimination among *Bacillus* species [31], B28 clustered with *B. siamensis* and separated from *B. amyloliquefaciens* (Figure 1B). In phylogenetic analysis of the eight housekeeping genes used for MLST, B28 grouped with *B. siamensis* (Figure S1). ANI values of the B28 genome sequence were 98.61%, 97.73%, 94.28%, and 94.06% with *B. siamensis* KCTC 13613^T^, *B. siamensis* SCSIO 05746, *B. velezensis* Y2, and *B. amyloliquefaciens* DSM7^T^, respectively. Thus, strain B28 was reclassified as *Bacillus siamensis*. 

### 3.2. General Genome Characteristics of Strain B28

The complete genome of *B. siamensis* strain B28 included a circular chromosome (3,946,178 bp) and two circular plasmids (Table 1). The G+C mol% of the genome of strain B28 was 45.85%. The genome contained 86 tRNA genes and 27 rRNA genes. The genome of strain B28 was bigger than that of the strain type KCTC 13613^T^ and smaller than that of strain SCSIO 05746, which was the only other complete genome of a *B. siamensis* strain available by 15 March 2021 (Table 1).

Analysis using COG functional categorization and SEED subsystem categorization, respectively, predicted 3573 and 1663 coding sequences (CDSs) in the genome of strain B28. COG analysis was revealed that amino acid transport and metabolism (291 genes, 8.14%) was the most abundant category, followed by transcription (266 genes, 7.44%) and carbohydrate transport and metabolism (214 genes, 5.99%) (Figure S2A). The order of abundance was similar in strain KCTC 13613^T^ and SCSIO 05746 (Figure S2A). 

In data from the SEED subsystem, 297 genes in the strain B28 genome (17.86%) were assigned to amino acid biosynthesis (Figure S2B). The next most abundant subsystem category was protein metabolism (211 genes, 12.69%), followed by carbohydrates (191 genes, 11.49%). These patterns were similar in the genome of strain KCTC 13613^T^ and SCSIO 05746.

Strain B28 possessed the two circular plasmids pB2801 (6.1 kb) and pB2802 (5.4 kb). Two replication protein genes (B28_04055 in p2801 and B28_04064 in pB2802), which are related to plasmid replication, were detected. However, most of the genes were annotated as hypothetical protein genes. 

To identify the unique genes of strain B28, we analyzed the shared genes between the genomes of B28 and SCSIO 05746, but not KCTC 13613^T^ as the genome is incomplete. The number of genes shared by the genomes of *B. siamensis* strains B28 and SCSIO 05746 is illustrated in a Venn diagram (Figure S3). The two strains share 3487 CDSs in their core genome, corresponding to approximately 91.2% and 84.0% of the CDSs in strains B28 and SCSIO 05746, respectively. The majority of strain-specific genes are associated with hypothetical proteins (Table S1). However, unique assigned CDSs of strain B28 included a relaxase, transposase, α-galactosidase, and triacylglycerol lipase. The α-galactosidase (EC 3.2.1.22; gene locus: JD965_RS14670) and triacylglycerol lipase (EC 3.1.1.3; JD965_RS01465) genes were located on the chromosome. α-Galactosidase is an exoglycosidase, which breaks terminal α-1,6 galactosidase bonds of melibiose, raffinose and polymeric galactomannans [36]. This enzyme contributes to the mitigation of intestinal discomfort in humans by improving the digestibility of carbohydrates such as melibiose and stachyose, which are contained in soybean foods [37]. Triacylglycerol lipase catalyzes the hydrolysis of triacylglycerol and generates free fatty acids, monoacylglycerol, diacylglycerol, and glycerol. This enzyme contributes to the enhancement of flavor during food fermentation [38]. Therefore, these genes in strain B28 may contribute to the enhancement of human health and the sensory properties of foods if the bacterium were used as a probiotic or fermentation starter, respectively.

### 3.3. Insights into Virulence of B. siamensis Strain B28

The food pathogen *B. cereus* produces several enterotoxins: three hemolytic enterotoxins, (NblA, NblC, and NblD), three nonhemolytic enterotoxins (NheA, NheB, and NheC), and one enterotoxin T (BcET) [35,39]. Although *B. siamensis* is a member of the same genus as notorious pathogenic *B. cereus*, *B. siamensis* is nonpathogenic. The absence of enterotoxin-encoding genes in *B. siamensis* strain B28 was verified by PCR (Figure S4). Moreover, no toxin-related genes were identified in the strain B28 genome. 

We also checked the antibiotic susceptibility of, and hemolysis by, strain B28. The strain was sensitive to chloramphenicol, clindamycin, erythromycin, gentamycin, lincomycin, streptomycin, tetracycline, and vancomycin, and did not exhibit β-hemolytic activity (Figure S4). 

The genome of strain B28 does encode a hemolysin-III like protein gene (*hlyIII*; JD965_RS10690). A homologous gene was also identified in *B. siamensis* strain SCSIO 05746, and in the closely related species *B. amyloliquefaciens* and *B. velezensis* (Table 2). Although hemolysis was verified for HlyIII from *Vibrio vulnificus* on overexpression in *E. coli* [40], its homolog in *Bacteriodes fragilis* was not linked to hemolytic activity [41]. Moreover, in our previous studies, the *hlyIII* gene was detected regardless of phenotypic hemolysis [42,43,44,45]. Therefore, we suggest that the *hlyIII* gene in *B. siamensis* strain B28 is not related to hemolysis. 

Putative efflux pumps or transporter genes for bicyclomycin, lincomycin, multiple drugs, and tetracycline were identified on the chromosomes of *B. siamensis* strains B28 and SCSIO 05746 (Table 2). However, strain B28 did not exhibit resistance to lincomycin or tetracycline (Figure S4). These genes have previously been identified in the genomes of several strains that did not show resistance to the respective antibiotics [15,42], and the genes were detected on the chromosomes of *B. amyloliquefaciens* and *B. velezensis*, not on plasmids. These results imply that these were not acquired antibiotic resistance genes (Table 2). Therefore, we suggest that the annotated chromosomally encoded antibiotic resistance genes in the B28 genome are not strain-specific and may not actually contribute to antibiotic resistance. 

Overall, phenotypic and genomic analyses suggest that *B. siamensis* strain B28 is a safe bacterium for use in food and medicine.

### 3.4. Probiotic Properties of B. siamensis Strain B28

The live microorganisms in probiotics have health benefits for humans and animals [46]. Generally, the requirements for probiotics are acid tolerance for passage through the gastric tract (to reach the intestine); the ability to survive in harsh conditions, including in manufacturing processes and the human/animal gut; adherence in the gut; antimicrobial activity against pathogenic bacteria; carbohydrate, lipid, and protein use; and health enhancement, such as enhancement of immunogenicity. 

#### 3.4.1. Survivability of Strain B28

The genome of *B. siamensis* strain B28 suggests that spore-forming ability, biofilm formation, and cholylglycine hydrolase (EC 3.5.1.24; a bile salt hydrolase) might aid survival of this strain in extreme conditions (Table S2). The B28 genome encodes spore-forming genes including spore wall synthetic proteins, as well as proteins for spore germination. Species that can form spores can withstand the harsh conditions used by the food processing industry, such as high temperatures. Biofilm-producing bacteria can also persist in extreme environments, and strain B28 possesses genes related to biofilm formation. These results indicate that *B. siamensis* strain B28 might reach the intestinal tract via biofilm formation, spore formation, and/or the deconjugation of bile salts.

#### 3.4.2. Adhesive Ability of Strain B28

Successful probiotics should be able to adhere in the gut [47]. Biofilms, exopolysaccharide (EPS), fibronectin, and flagella are factors related to adhesion [48,49,50,51]. The B28 genome suggests that EPS can be produced during biofilm formation (Table S2). It also contains genes related to fibronectin biosynthesis and flagellum formation.

#### 3.4.3. Antibacterial Activity

**Figure 2:**
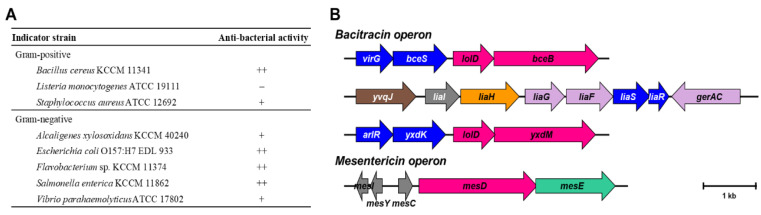
Antibacterial activity (**A**) and annotated bacteriocin-related genes (**B**) of *B. siamensis* strain B28.

The antibacterial activity of *B. siamensis* strain B28 against eight foodborne pathogens was evaluated via an agar plate diffusion method. The strain inhibited the growth of the Gram-positive strains *B. cereus* and *S. aureus* and the Gram-negative strains *Alcaligenes xylosoxidans*, *E. coli*, *Flavobacterium*, *Salmonella enterica*, and *Vibrio parahaemolyticus* (Figure 2).

The B28 genome includes two bacteriocin-related operons—the bacitracin operon and the mesentericin operon (Figure 2). Bacitracin is a branched cyclic dodecylpeptide antibiotic synthesized nonribosomaly [52] that interferes with cell wall synthesis [53]. The bacitracin operon of strain B28 includes genes encoding a small membrane protein (*liaI*), phage shock protein (*liaH*), antibacterial peptide (*liaG*), transporter (*liaF*), sensor kinase (*liaS*), and response regulator (*liaR*) (Figure 2). Mesentericin is a small non-lantibiotic bacteriocin that shows bactericidal activity [54]. These gene sets might confer the phenotypical antibacterial activities of strain B28. Bacteriocin-producing bacteria have an advantage in the gut. These results suggest that strain B28 is a potential probiotic with antimicrobial activity.

#### 3.4.4. Potential for Health Enhancement by Strain B28

**Figure 3:**
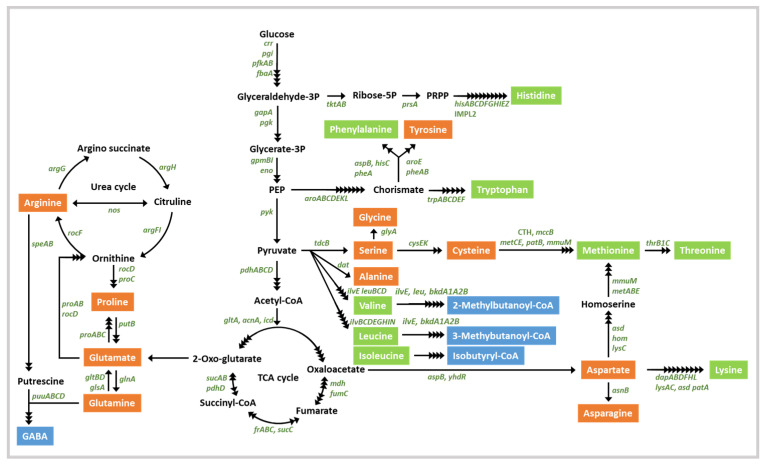
Predicted amino acid synthesis pathways in *B. siamensis* strain B28. Enzyme-encoding genes are displayed in green. Essential amino acids, non-essential amino acids, and other metabolites are green, orange, and blue-boxed, respectively. The black arrows indicate potential enzymatic reactions.

The strain B28 genome indicates the ability to supply the essential amino acids, isoleucine, leucine, lysine, methionine, phenylalanine, threonine, tryptophan, and valine, in the intestine via synthesis from glucose (Table S3 and Figure 3). Strain B28 also possesses genes for the production of γ-aminobutyric acid (GABA) from glutamate and branched chain fatty acids (BCFAs) from isoleucine, leucine, and valine (Figure 3). GABA and BCFAs are bioactive compounds that are important for human health; for example, they show anti-proliferative activity [55,56]. Genomic analysis also revealed that strain B28 encodes two gamma-glutamyltransferase (GGT) genes (EC 2.3.2.2; JD965_RS09945 and JD965_RS17695) involved in the production of γ-glutamyl peptide from free amino acids; γ-glutamyl peptide has beneficial effects in human tissue, such as antioxidation and anticancer activities [57]. The *B. siamensis* B28 genome possesses a subtilisin-encoding gene (EC 3.4.21.62; nattokinase; JD965_RS05730); subtilisin shows antithrombotic and antihypertensive effects [58]. These analyses suggest that strain B28 might supply essential amino acids and bioactive compounds to a human host through the gut.

**Figure 4:**
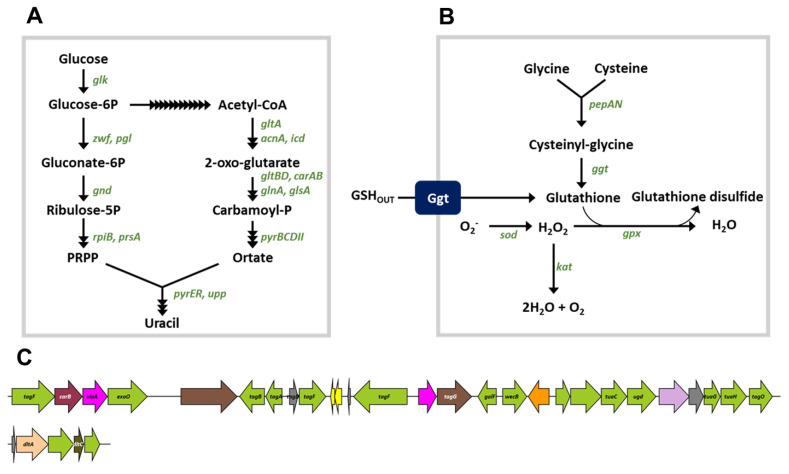
Synthesis pathways of uracil (**A**) and glutathione (**B**), and operon for lipoteichoic acid synthesis (**C**) in *B. siamensis* strain B28. Potential enzyme-encoding genes are displayed in green letters. The black arrows indicate potential enzymatic reactions in the B28 genome.

Previously, *B. siamensis* strain B28 was found to inhibit the expression of microphthalmia-associated transcription factor and β-catenin in human embryonic kidney 293 cells. β-catenin is important for carcinogenesis and tumor progression. We assumed that the effect on microphthalmia-associated transcription factor contributed to the whitening effects of strain B28 [19]. To find the genetic basis for these effects, we searched the strain B28 genome for tyrosinase inhibitors (for the whitening effect) and regulators of β-catenin expression. Tyrosinase plays a key role in melanogenesis in mammals, and bacteria produce tyrosinase inhibitors during fermentation [59]. The B28 genome possesses genes for uracil, lipoteichoic acid, and glutathione synthesis; these compounds are tyrosinase inhibitors found in microorganisms [59,60] (Figure 4 and Table S4). Moreover, glutathione is involved in the scavenging of reactive oxygen species, which induces β-catenin expression [61]; the B28 genome also encodes several enzymes (catalase, peroxidase, and superoxidase dismutase) that scavenge reactive oxygen species (Table S4). Additionally, glycogen synthase kinase-3 (EC 2.7.11.1) activity could affect β-catenin expression, and the B28 genome contains eight genes for the production of glycogen synthase kinase-3 [62] (Table S4).

*Bacillus siamensis* was registered in 2010 [63] and the predominant species in fermented foods [64,65]. *Bacillus siamensis* showed the enzymatic activities which conferred the enhancement of sensory properties [64,65]. Therefore, *B. siamensis* was suggested as a starter candidate species for food fermentation. Our current experimental results provide genomic insights into *B. siamensis* strain B28 and suggest that it lacks acquired antibiotic resistance, hemolysin, and enterotoxin genes. The genome indicates that strain B28 possesses genes for survival in harsh conditions, adhesion, antibacterial activity against foodborne pathogens, essential amino acid synthesis, and the enhancement of human health. These results suggested the possibility of using *B. siamensis* strain B28 as a safe probiotic strain. *B. siamensis* strain B28 could be developed for use as a valuable food by the functional food, feed, and medical industries.

## Figures and Tables

**Table 1:** Genome features of *Bacillus siamensis* strains.

Feature	B28	SCSIO 05746	KCTC 13613^T^
Size (bp)	3,957,728	4,280,711	3,779,696
Chromosome size (bp)	3,946,178	4,268,316	-
G+C content (mol%)	45.85	45.99	46.30
No. of plasmids	2 ^a^	1 ^b^	-
Open reading frames	4034	4375	3839
CDSs assigned by COG ^c^	3573	3706	3451
CDSs assigned by SEED ^d^	1663	1724	1617
No. of rRNAs	27	27	3
No. of tRNAs	86	86	78
Contigs	3	2	51
Scaffolds	0	0	51
Origin	*Kimchi*	Sea mud	Salted crab
References	Complete	Complete	Draft

^a^ Plasmids in strain B28: pB2801, 6.1 kb, and pB2802, 5.4 kb; ^b^ Plasmids in strain SCSIO 05746: pSCSIO05746, 12.4 kb; ^c^ COG results were retrieved from EZBioCloud data: https://www.ezbiocloud.net/ accessed on June 2021; ^d^ SEED results were retrieved from RAST data: https://rast.nmpdr.org/rast.cgi accessed on June 2021. CDSs, coding sequences; COG, Clusters of Orthologous Groups.

**Table 2:** Potential virulence determinants identified in *Bacillus siamensis* and genomes of related species.

Gene Locus	Product	Gene ^a^	KEEG ^b^	COG	Presence of Gene in *Bacillus* Genomes				
					*B. siamensis*	*B. amyloliquefaciens*		*B. velezensis*	
					SCSIO 05746	FS1092	RD7-7	JJ-D34	KMU01
**Hemolysis-related**									
JD965_RS10690	Hemolysin-3 like protein	*hlyIII*	K11068	S	●	●	●	●	●
**Antibiotic resistance**									
JD965_RS01450	Lincomycin resistance protein	*lmrB*	K18926	P	●	●	●	●	●
JD965_RS01610	Multidrug resistance protein		K18935	P	●	●	●	●	●
JD965_RS02265	Small multidrug resistance pump	*smr*	K03297	P	●	●	-	●	●
JD965_RS05435	ATP-binding cassette, subfamily B, multidrug efflux pump	*mdlA, smdA*	K18889	V	●	●	●	●	●
JD965_RS05440	ATP-binding cassette, subfamily B, multidrug efflux pump	*mdlB, smdA*	K18890	V	●	●	●	●	●
JD965_RS05865	MFS transporter	*blt*	K08153	G	●	●	●	●	●
JD965_RS07140	Paired small multidrug resistance pump	*ykkC*	K18924	P	●	●	●	●	●
JD965_RS07145	Paired small multidrug resistance pump	*ykkD*	K18925	P	●	●	●	●	●
JD965_RS09245	Small multidrug resistance pump	*smr*	K03297	P	●	●	●	●	●
JD965_RS09240	Multidrug resistance protein		K11815	P	●	●	●	●	●
JD965_RS09920	Probable multidrug resistance protein		K03327	V	●	●	●	●	●
JD965_RS10260	Multidrug resistance protein, MATE family	*norM*	K03327	V	●	●	●	●	●
JD965_RS12840	Tetracycline resistance protein	*tetB*	K08168	G	●	●	-	●	●
JD965_RS18410	Bicyclomycin resistance protein	*bcr, tcaB*	K07552	P	●	●	●	●	●

^a^ Gene (BlastKOALA); ^b^ KEGG (The Kyoto Encyclopedia of Genes and Genomes number is from the KEGG orthology database (http://www.genome.jp accessed on June 2021). - indicates that a gene was not identified in the strain.

## Data Availability

The data presented in this study are available in the article.

## Supplementary Materials

The following are available online at https://www.mdpi.com/article/10.3390/foods10081906/s1, Figure S1: Phylogenetic trees based on the entire gene sequences of adk, ccpA, glpF, gmk, ilvD, pur, spo0A, and tpi showing the relationships between strain B28 and other Bacillus spp. The phylogenetic trees were constructed using the maximum likelihood method. Branches with bootstrap values <50% were collapsed. The distance scales are shown under the trees. Figure S2: Comparison of functional categories of genes in three Bacillus siamensis genomes based on Clusters of Orthologous Groups (COG) (A) and SEED (B) analyses. Genome sequences of B. siamensis strains B28 and SCSIO 05746 were uploaded to the COG and SEED viewer servers independently. Functional roles of annotated genes were assigned and grouped by subsystem feature categories. Colored bars indicate the number of genes assigned to each category. Figure S3: Venn diagram comparing the two B. siamensis genomes. The Venn diagram enumerates the pan-genome of strains B28 and SCSIO 05746 generated using EDGAR (the Efficient Database framework for comparative Genome Analyses using BLASTP score Ratios). The overlapping region represents common coding sequences (CDSs) shared between the B. siamensis genomes. The numbers outside the overlapping regions indicate the numbers of CDSs in each genome without homologs in the other genome. Figure S4: Test of the antibiotic susceptibility of B. siamensis strain B28 (A); hemolysis by strain B28 (B); and the presence of enterotoxin genes in strain B28 (C). Table S1: List of singletons generated by comparing the genomes of two B. siamensis strains Table S2: List of genes in strain B28 related to probiotic properties Table S3: List of genes in strain B28 related to amino acid synthesis Table S4: List of genes in strain B28 related to whitening and scavenging of reactive oxygen species.
