# Supplementary material for: Functional Genomic Insights into Probiotic Bacillus siamensis Strain B28 from Traditional Korean Fermented Kimchi
Source: Foods. 2021 Aug 17;10(8):1906. doi: 10.3390/foods10081906 (PMC8394110; doi:10.3390/foods10081906)
Supplement: Supplementary file 1 [file foods-10-01906-s001.zip › foods-1288651-supplementary.pdf]

Figure S1. Phylogenetic analysis of eight housekeeping genes, *adk*, *ccpA*, *glpF*, *gmk*, *ilvD*, *pur*, *spo0A*, and *tpi*. The phylogenetic trees were developed using the maximum likelihood method with 1,000 bootstrap values. Branches with bootstrap values lower than 50% were collapsed.

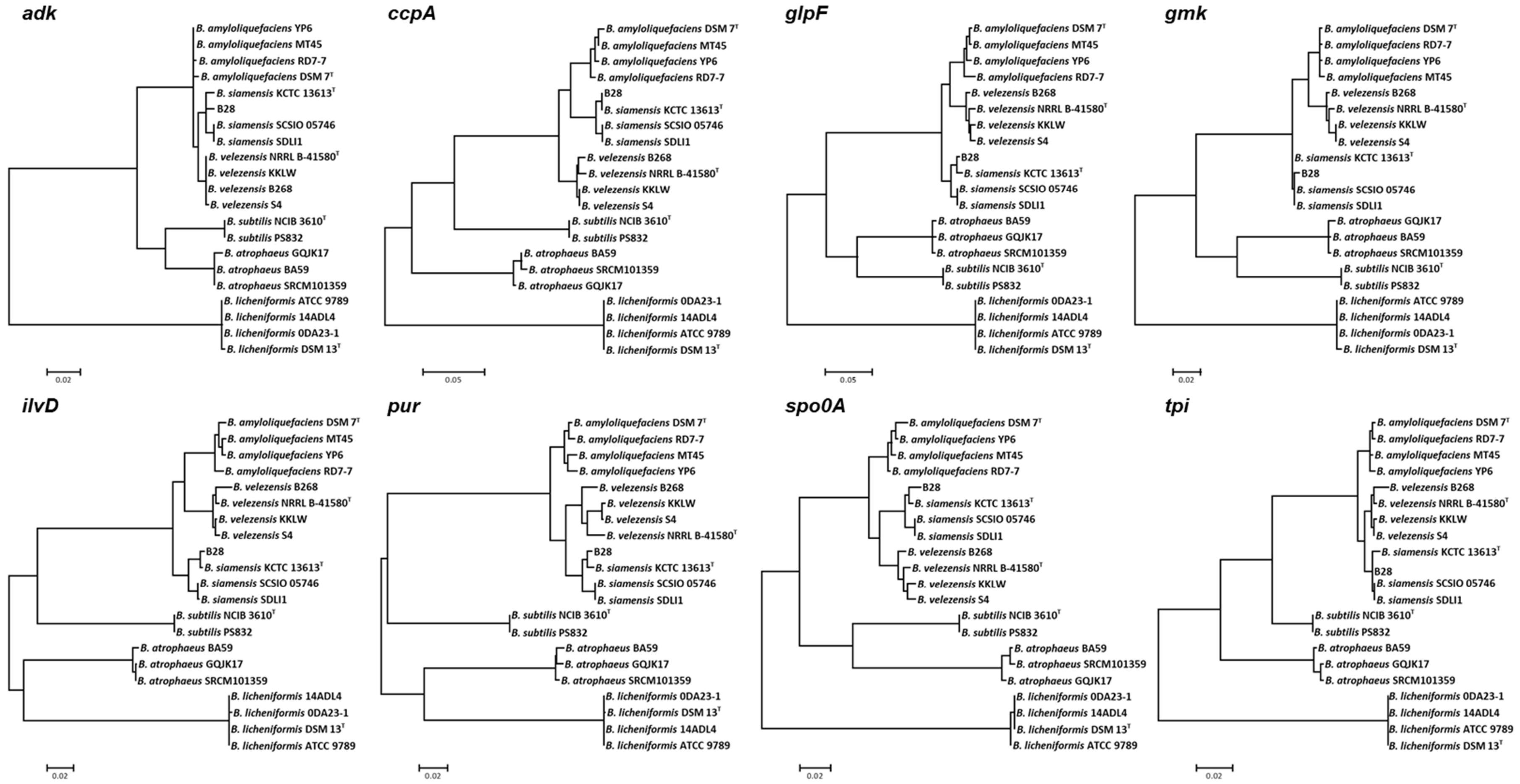

Figure S2. Comparison of functional categories of genes in three *Bacillus siamensis* genomes based on Clusters of Orthologous Groups (COG) (A) and SEED (B) analyses. Genome sequences of *B. siamensis* strains B28 and SCSIO 05746 were uploaded to the COG and SEED viewer servers independently. Functional roles of annotated genes were assigned and grouped by subsystem feature categories. Colored bars indicate the number of genes assigned to each category.

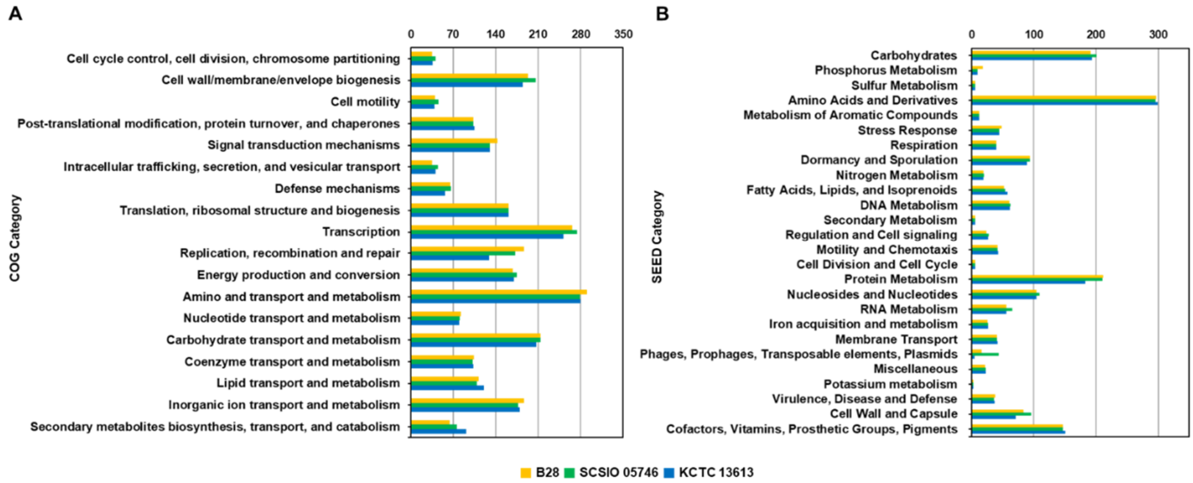

Figure S3. Venn diagram comparing the two *B. siamensis* genomes. The Venn diagram enumerates the pan-genome of strains B28 and SCSIO 05746 generated using EDGAR (the Efficient Database framework for comparative Genome Analyses using BLASTP score Ratios). The overlapping region represents common coding sequences (CDSs) shared between the *B. siamensis* genomes. The numbers outside the overlapping regions indicate the numbers of CDSs in each genome without homologs in the other genome.

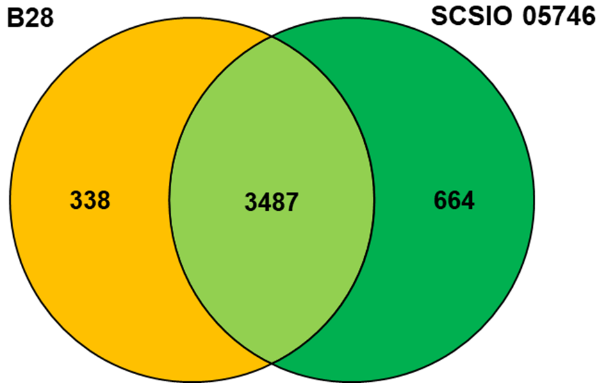

Figure S4. Tests of the antibiotic susceptibility of *B. siamensis* strain B28 (A); hemolysis by strain B28 (B); and the amplicon of seven enterotoxin genes (C)

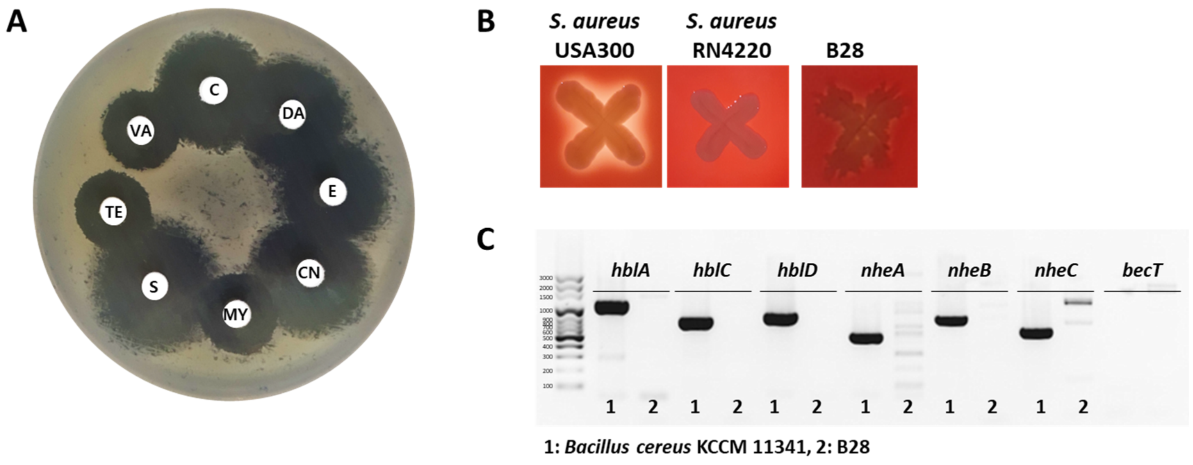

Ab

Discs: C, chloramphenicol (30 µg); CN, gentamycin (30 µg); DA, clindamycin (10 µg); E, erythromycin (15 µg); MY, lincomycin (15 µg); S, streptomycin (300 µg); Te, tetracycline (30 µg); VA, vancomycin (30 µg).

Table S1. List of singletons generated by comparing the genomes of two *B. siamensis* strains.

Strain	Product	Gene locus
B28		
	Hypothetical protein	JD965_RS00100
	ABC transporter ATP-binding protein	JD965_RS01150
	ABC-2 transporter permease	JD965_RS01155
	Alpha/beta hydrolase	JD965_RS01180
	HAMP domain-containing histidine kinase	JD965_RS01190
	Protein kinase	JD965_RS01195
	Phosphotransferase	JD965_RS01380
	Tryptophan RNA-binding attenuator protein inhibitory protein	JD965_RS01385
	Triacylglycerol lipase	JD965_RS01465
	MsnO8 family LLM class oxidoreductase	JD965_RS01530
	ArpU family transcriptional regulator	JD965_RS02215
	Hypothetical protein	JD965_RS02225
	Hypothetical protein	JD965_RS02515
	EamA family transporter	JD965_RS02525
	Hypothetical protein	JD965_RS02535
	Hypothetical protein	JD965_RS02540
	Oxygenase	JD965_RS02590
	GntR family transcriptional regulator	JD965_RS02650
	PTS lactose/cellobiose transporter subunit IIA	JD965_RS02655
	PTS sugar transporter subunit IIB	JD965_RS02660
	Beta-glucosidase	JD965_RS02665
	PTS transporter subunit EIIC	JD965_RS02670
	ROK family protein	JD965_RS02675
	Histidinol-phosphate transaminase	JD965_RS02685
	Amino acid permease	JD965_RS02690
	Molybdopterin-dependent oxidoreductase	JD965_RS02695
	SUMF1/EgtB/PvdO family nonheme iron enzyme	JD965_RS02700
	DUF2000 domain-containing protein	JD965_RS02705
	PLP-dependent aminotransferase family protein	JD965_RS02715
	IS3 family transposase	JD965_RS02765
	NAD(P)/FAD-dependent oxidoreductase	JD965_RS02790
	TetR/AcrR family transcriptional regulator	JD965_RS02875
	GntR family transcriptional regulator	JD965_RS03030
	ImmA/IrrE family metallo-endopeptidase	JD965_RS03195
	Helix-turn-helix domain-containing protein	JD965_RS03210
	Helix-turn-helix transcriptional regulator	JD965_RS03215
	Hypothetical protein	JD965_RS03220
	Hypothetical protein	JD965_RS03225
	Helix-turn-helix transcriptional regulator	JD965_RS03230
	Helix-turn-helix domain-containing protein	JD965_RS03235
	Phage antirepressor KilAC domain-containing protein	JD965_RS03240

Strain	Product	Gene locus
	Hypothetical protein	JD965_RS03255
	ATP-binding protein	JD965_RS03285
	RusA family crossover junction endodeoxyribonuclease	JD965_RS03295
	Hypothetical protein	JD965_RS03325
	Hypothetical protein	JD965_RS03340
	Hypothetical protein	JD965_RS03345
	Hypothetical protein	JD965_RS03350
	Hypothetical protein	JD965_RS03355
	Hypothetical protein	JD965_RS03375
	Type II toxin-antitoxin system PemK/MazF family toxin	JD965_RS03380
	Hypothetical protein	JD965_RS03385
	Hypothetical protein	JD965_RS03415
	Right-handed parallel beta-helix repeat-containing protein	JD965_RS03485
	BppU family phage baseplate upper protein	JD965_RS03490
	N-acetylmuramoyl-L-alanine amidase	JD965_RS03510
	Hypothetical protein	JD965_RS03515
	5-aminolevulinate synthase	JD965_RS03520
	DMT family transporter	JD965_RS03525
	Class I tRNA ligase family protein	JD965_RS03530
	Cupin domain-containing protein	JD965_RS03535
	YqcI/YcgG family protein	JD965_RS03540
	GNAT family N-acetyltransferase	JD965_RS03545
	FAD-dependent oxidoreductase	JD965_RS03550
	MFS transporter	JD965_RS03555
	IS1182 family transposase	JD965_RS03605
	IS3 family transposase	JD965_RS03640
	ATP-grasp domain-containing protein	JD965_RS03685
	ATP-grasp domain-containing protein	JD965_RS03690
	Isocitrate lyase/phosphoenolpyruvate mutase family protein	JD965_RS03695
	Aminotransferase class I/II-fold pyridoxal phosphate-dependent enzyme	JD965_RS03700
	MFS transporter	JD965_RS03705
	IS3 family transposase	JD965_RS03865
	ATP-binding cassette domain-containing protein	JD965_RS03910
	ABC transporter permease	JD965_RS03915
	ABC transporter ATP-binding protein	JD965_RS03920
	ABC transporter permease	JD965_RS03925
	MepB family protein	JD965_RS03970
	Sulfate ABC transporter substrate-binding protein	JD965_RS03990
	Sulfate ABC transporter permease subunit CysT	JD965_RS03995
	Sulfate ABC transporter permease subunit CysW	JD965_RS04000
	TOBE-like domain-containing protein	JD965_RS04005
	Hypothetical protein	JD965_RS04045
	Transcriptional regulator	JD965_RS04050
	ATP-binding cassette domain-containing protein	JD965_RS04055

Strain	Product	Gene locus
	ABC transporter permease	JD965_RS04060
	Hypothetical protein	JD965_RS04120
	Hypothetical protein	JD965_RS04125
	Hypothetical protein	JD965_RS04130
	Hypothetical protein	JD965_RS04135
	Hypothetical protein	JD965_RS04140
	Hypothetical protein	JD965_RS04145
	Hypothetical protein	JD965_RS04150
	Hypothetical protein	JD965_RS04155
	Hypothetical protein	JD965_RS04160
	Helix-turn-helix transcriptional regulator	JD965_RS04170
	DUF1700 domain-containing protein	JD965_RS04175
	Lactococcin 972 family bacteriocin	JD965_RS04180
	DUF1430 domain-containing protein	JD965_RS04185
	ATP-binding cassette domain-containing protein	JD965_RS04190
	Bacteriocin-like WGxF protein	JD965_RS04195
	Hypothetical protein	JD965_RS04200
	Hypothetical protein	JD965_RS04205
	MerR family transcriptional regulator	JD965_RS04210
	GNAT family N-acetyltransferase	JD965_RS04245
	Spore surface glycoprotein BclB	JD965_RS04285
	Citrate transporter	JD965_RS04305
	MBL fold metallo-hydrolase	JD965_RS04310
	ATP-binding cassette domain-containing protein	JD965_RS04485
	ABC transporter permease	JD965_RS04490
	Phosphatase PAP2 family protein	JD965_RS04495
	Response regulator transcription factor	JD965_RS04500
	HAMP domain-containing histidine kinase	JD965_RS04505
	IS1182 family transposase	JD965_RS04665
	Hypothetical protein	JD965_RS04915
	Hypothetical protein	JD965_RS04920
	MATE family efflux transporter	JD965_RS04925
	Methyltransferase domain-containing protein	JD965_RS04930
	Hypothetical protein	JD965_RS04935
	Hypothetical protein	JD965_RS04945
	Spore coat protein	JD965_RS04950
	Hypothetical protein	JD965_RS05155
	Response regulator transcription factor	JD965_RS05260
	Hypothetical protein	JD965_RS05680
	Hypothetical protein	JD965_RS05790
	IS1182 family transposase	JD965_RS05805
	IS1182 family transposase	JD965_RS05810
	YbjQ family protein	JD965_RS05815
	IS1182 family transposase	JD965_RS05855

Strain	Product	Gene locus
	GNAT family N-acetyltransferase	JD965_RS06000
	FosM family fosfomycin resistance protein	JD965_RS06065
	IS1182 family transposase	JD965_RS06120
	Hypothetical protein	JD965_RS06130
	IS3 family transposase	JD965_RS06245
	Subclass B1 metallo-beta-lactamase	JD965_RS06430
	DUF1878 family protein	JD965_RS06500
	Hypothetical protein	JD965_RS06505
	Hypothetical protein	JD965_RS06510
	Hypothetical protein	JD965_RS06515
	Hypothetical protein	JD965_RS06520
	IS3 family transposase	JD965_RS06525
	Hypothetical protein	JD965_RS06530
	ImmA/IrrE family metallo-endopeptidase	JD965_RS06535
	Helix-turn-helix domain-containing protein	JD965_RS06540
	YolD-like family protein	JD965_RS06545
	Hypothetical protein	JD965_RS06560
	Hypothetical protein	JD965_RS06565
	UPF0715 family protein	JD965_RS06580
	HIT domain-containing protein	JD965_RS06590
	Hypothetical protein	JD965_RS06595
	DUF3147 family protein	JD965_RS06670
	MarR family transcriptional regulator	JD965_RS06675
	Hypothetical protein	JD965_RS06760
	Winged helix-turn-helix domain-containing protein	JD965_RS06770
	HAMP domain-containing histidine kinase	JD965_RS06775
	Mas-related G-protein coupled receptor member D	JD965_RS06780
	IS1182 family transposase	JD965_RS06805
	N-acetylmuramoyl-L-alanine amidase	JD965_RS06810
	Hypothetical protein	JD965_RS06985
	Transcriptional regulator YeiL	JD965_RS07210
	Hypothetical protein	JD965_RS07465
	IS1182 family transposase	JD965_RS07690
	IS1182 family transposase	JD965_RS07855
	IS1182 family transposase	JD965_RS08065
	IS1182 family transposase	JD965_RS09095
	DUF3902 family protein	JD965_RS09425
	DUF4944 domain-containing protein	JD965_RS09430
	GNAT family N-acetyltransferase	JD965_RS09455
	YndJ family transporter	JD965_RS09660
	Hypothetical protein	JD965_RS09880
	Hypothetical protein	JD965_RS09905
	Sugar kinase	JD965_RS10025
	Hypothetical protein	JD965_RS10055

Strain	Product	Gene locus
	EamA family transporter RarD	JD965_RS10285
	Tautomerase family protein	JD965_RS10300
	Hypothetical protein	JD965_RS10305
	DUF3221 domain-containing protein	JD965_RS10500
	Hypothetical protein	JD965_RS10515
	Pentapeptide repeat-containing protein	JD965_RS10520
	Hypothetical protein	JD965_RS10525
	Tyrosine-type recombinase/integrase	JD965_RS10530
	Hypothetical protein	JD965_RS10560
	Hypothetical protein	JD965_RS10580
	LysR family transcriptional regulator	JD965_RS10585
	Pyridoxal-phosphate dependent enzyme	JD965_RS10590
	Pyridoxal-phosphate dependent enzyme	JD965_RS10595
	Hypothetical protein	JD965_RS10605
	Tetratricopeptide repeat protein	JD965_RS10610
	Thermonuclease family protein	JD965_RS10620
	IS3 family transposase	JD965_RS10755
	Amino acid adenylation domain-containing protein	JD965_RS11770
	Amino acid adenylation domain-containing protein	JD965_RS11775
	Hypothetical protein	JD965_RS11780
	Beta-ketoacyl-ACP synthase II	JD965_RS11785
	Acyl carrier protein	JD965_RS11790
	SDR family oxidoreductase	JD965_RS11795
	Aromatic amino acid lyase	JD965_RS11800
	AMP-binding protein	JD965_RS11805
	Heme o synthase	JD965_RS11810
	IS3 family transposase	JD965_RS12320
	IS3 family transposase	JD965_RS12420
	LysR family transcriptional regulator	JD965_RS12790
	Amino acid permease	JD965_RS12795
	Cof-type HAD-IIB family hydrolase	JD965_RS12865
	IS1182 family transposase	JD965_RS13055
	IS1182 family transposase	JD965_RS13190
	Hypothetical protein	JD965_RS13355
	Hypothetical protein	JD965_RS13395
	Hypothetical protein	JD965_RS13485
	Hypothetical protein	JD965_RS13495
	Hypothetical protein	JD965_RS13500
	Hypothetical protein	JD965_RS13505
	Hypothetical protein	JD965_RS13520
	Hypothetical protein	JD965_RS13560
	Helix-turn-helix transcriptional regulator	JD965_RS13565
	Helix-turn-helix transcriptional regulator	JD965_RS13570
	ImmA/IrrE family metallo-endopeptidase	JD965_RS13575

Strain	Product	Gene locus
	Recombinase family protein	JD965_RS13580
	Family 43 glycosylhydrolase	JD965_RS13995
	PhrK family phosphatase-inhibitory pheromone	JD965_RS14155
	Hypothetical protein	JD965_RS14160
	DUF2992 family protein	JD965_RS14165
	Peptidoglycan-binding protein	JD965_RS14170
	IS1182 family transposase	JD965_RS14175
	IS3 family transposase	JD965_RS14280
	PhrK family phosphatase-inhibitory pheromone	JD965_RS14385
	Hypothetical protein	JD965_RS14395
	Substrate-binding domain-containing protein	JD965_RS14650
	Extracellular solute-binding protein	JD965_RS14655
	ABC transporter permease subunit	JD965_RS14660
	ABC transporter permease subunit	JD965_RS14665
	Alpha-galactosidase MelA	JD965_RS14670
	Hypothetical protein	JD965_RS15140
	Hypothetical protein	JD965_RS15150
	Transporter	JD965_RS15260
	NAD(P)H-dependent oxidoreductase	JD965_RS15270
	Competence pheromone ComX	JD965_RS15445
	IS1182 family transposase	JD965_RS15630
	Hypothetical protein	JD965_RS15940
	Hypothetical protein	JD965_RS15945
	Chitosanase	JD965_RS15955
	LysR family transcriptional regulator	JD965_RS16095
	Hypothetical protein	JD965_RS16255
	DUF4885 family protein	JD965_RS16365
	Hypothetical protein	JD965_RS16370
	Hypothetical protein	JD965_RS16380
	IS3 family transposase	JD965_RS16385
	ABC transporter permease	JD965_RS16605
	Sugar porter family MFS transporter	JD965_RS16695
	N-acetylglucosamine-6-phosphate deacetylase	JD965_RS16725
	LacI family DNA-binding transcriptional regulator	JD965_RS16730
	Permease	JD965_RS16735
	Cof-type HAD-IIB family hydrolase	JD965_RS16740
	IS3 family transposase	JD965_RS16760
	Hypothetical protein	JD965_RS16770
	Hypothetical protein	JD965_RS16775
	Hypothetical protein	JD965_RS16780
	IS3 family transposase	JD965_RS17175
	CDP-glycerol glycerophosphotransferase family protein	JD965_RS17470
	Glycosyltransferase	JD965_RS17505
	DegT/DnrJ/EryC1/StrS family aminotransferase	JD965_RS17510

Strain	Product	Gene locus
	ATP-grasp domain-containing protein	JD965_RS17515
	CDP-glycerol glycerophosphotransferase family protein	JD965_RS17520
	GDP-mannose 4,6-dehydratase	JD965_RS17525
	YncE family protein	JD965_RS18255
	Helix-turn-helix domain-containing protein	JD965_RS18270
	Hypothetical protein	JD965_RS18275
	DUF4177 domain-containing protein	JD965_RS18280
	Helix-turn-helix domain-containing protein	JD965_RS18390
	IS3 family transposase	JD965_RS18400
	Sensor histidine kinase	JD965_RS18435
	Response regulator transcription factor	JD965_RS18440
	ABC transporter permease	JD965_RS18450
	ABC transporter ATP-binding protein	JD965_RS18455
	YafY family transcriptional regulator	JD965_RS18600
	SDR family oxidoreductase	JD965_RS18605
	Hypothetical protein	JD965_RS18610
	Dabb family protein	JD965_RS18615
	Hypothetical protein	JD965_RS18710
	Hypothetical protein	JD965_RS19035
	Hypothetical protein	JD965_RS19040
	Hypothetical protein	JD965_RS19045
	Hypothetical protein	JD965_RS19050
	PTS glucose transporter subunit IIA	JD965_RS19080
	DUF3427 domain-containing protein	JD965_RS19085
	(deoxy)nucleoside triphosphate pyrophosphohydrolase	JD965_RS19090
	HAMP domain-containing histidine kinase	JD965_RS19180
	Response regulator	JD965_RS19185
	ABC transporter ATP-binding protein	JD965_RS19190
	ABC transporter permease	JD965_RS19195
	ABC transporter permease	JD965_RS19200
	Hypothetical protein	JD965_RS19205
	Helix-turn-helix transcriptional regulator	JD965_RS19300
	Hypothetical protein	JD965_RS19305
	Blp family class II bacteriocin	JD965_RS19310
	Hypothetical protein	JD965_RS19315
	Peptidase domain-containing ABC transporter	JD965_RS19320
	HlyD family efflux transporter periplasmic adaptor subunit	JD965_RS19325
	Hypothetical protein	JD965_RS19425
	Hypothetical protein	JD965_RS19430
	Hypothetical protein	JD965_RS19435
	Hypothetical protein	JD965_RS19440
	Deoxyguanosinetriphosphate triphosphohydrolase	JD965_RS19445
	Cupin-like domain-containing protein	JD965_RS19515
	ABC-F family ATP-binding cassette domain-containing protein	JD965_RS19520

Strain	Product	Gene locus
	MFS transporter	JD965_RS19525
	ABC transporter substrate-binding protein	JD965_RS19530
	ThiF family adenylyltransferase	JD965_RS19535
	IS3 family transposase	JD965_RS19695
	Hypothetical protein	JD965_RS19700
	DUF5068 domain-containing protein	JD965_RS19705
	DMT family transporter	JD965_RS19740
	Response regulator transcription factor	JD965_RS19745
	Anthranilate synthase component I	JD965_RS19750
	DMT family transporter	JD965_RS19755
	HD domain-containing protein	JD965_RS19800
	DUF4367 domain-containing protein	JD965_RS19820
	Hypothetical protein	JD965_RS19825
	Hypothetical protein	JD965_RS19895
	DUF3850 domain-containing protein	JD965_RS19900
	Hypothetical protein	JD965_RS19945
	Hypothetical protein	JD965_RS19950
	Hypothetical protein	JD965_RS19955
	Protein rep	JD965_RS20100*
	Hypothetical protein	JD965_RS20105*
	Hypothetical protein	JD965_RS20110*
	Plasmid recombination protein	JD965_RS20115*
	DUF3967 domain-containing protein	JD965_RS20120*
	Hypothetical protein	JD965_RS20125*
	Hypothetical protein	JD965_RS20130*
	Hypothetical protein	JD965_RS20135*
	Hypothetical protein	JD965_RS20140*
	Hypothetical protein	JD965_RS20145*
	Protein rep	JD965_RS20150*
	Hypothetical protein	JD965_RS20155*
	Hypothetical protein	JD965_RS20160*
	Relaxase/mobilization nuclease domain-containing protein	JD965_RS20165*
	Hypothetical protein	JD965_RS20170*
SCSIO 05746		
	Type VI secretion system contractile sheath protein Tss	CWD84_RS22230
	Type VI secretion system contractile sheath protein Tss	CWD84_RS22255
	Type VI secretion system contractile sheath protein Tss	CWD84_RS22280
	Type VI secretion system contractile sheath protein TssC	CWD84_RS22305
	IS3 family transposase	CWD84_RS03855
	Site-specific integrase	CWD84_RS07550
	AimR family lysis-lysogeny pheromone receptor	CWD84_RS07555
	Helix-turn-helix transcriptional regulator	CWD84_RS07560
	Helix-turn-helix transcriptional regulator	CWD84_RS07565
	Hypothetical protein	CWD84_RS07570

Strain	Product	Gene locus
	Conserved phage C-terminal domain-containing protein	CWD84_RS07575
	ATP-binding protein	CWD84_RS07580
	Hypothetical protein	CWD84_RS07585
	XtrA/YqaO family protein	CWD84_RS07590
	Hypothetical protein	CWD84_RS07595
	Hypothetical protein	CWD84_RS07600
	Hypothetical protein	CWD84_RS07605
	DNA adenine methylase	CWD84_RS07610
	Hypothetical protein	CWD84_RS07615
	DUTP diphosphatase	CWD84_RS07620
	Hypothetical protein	CWD84_RS07625
	Hypothetical protein	CWD84_RS07630
	Hypothetical protein	CWD84_RS07635
	Hypothetical protein	CWD84_RS07640
	Hypothetical protein	CWD84_RS07645
	ArpU family transcriptional regulator	CWD84_RS07650
	Tyrosine-type recombinase/integrase	CWD84_RS07655
	Cell division protein FtsK	CWD84_RS07660
	Hypothetical protein	CWD84_RS07670
	Hypothetical protein	CWD84_RS07675
	Hypothetical protein	CWD84_RS07680
	Hypothetical protein	CWD84_RS07685
	HNH endonuclease	CWD84_RS07690
	Phage terminase small subunit P27 family	CWD84_RS07700
	Terminase large subunit	CWD84_RS07705
	Phage portal protein	CWD84_RS07710
	HK97 family phage prohead protease	CWD84_RS07715
	Phage major capsid protein	CWD84_RS07720
	Collagen-like protein	CWD84_RS07725
	Phage gp6-like head-tail connector protein	CWD84_RS07730
	Phage head closure protein	CWD84_RS07735
	HK97 gp10 family phage protein	CWD84_RS07740
	DUF3168 domain-containing protein	CWD84_RS07745
	Tail protein	CWD84_RS07750
	Hypothetical protein	CWD84_RS07755
	Phage tail tape measure protein	CWD84_RS07760
	Phage tail family protein	CWD84_RS07765
	Phage tail protein	CWD84_RS07770
	Peptidase G2	CWD84_RS07775
	BppU family phage baseplate upper protein	CWD84_RS07780
	Hypothetical protein	CWD84_RS07785
	XkdX family protein	CWD84_RS07790
	Phage holin family protein	CWD84_RS07795
	N-acetylmuramoyl-L-alanine amidase	CWD84_RS07800

Strain	Product	Gene locus
	Hypothetical protein	CWD84_RS07805
	Hypothetical protein	CWD84_RS07810
	Hypothetical protein	CWD84_RS07815
	Hypothetical protein	CWD84_RS07820
	Hypothetical protein	CWD84_RS07825
	Site-specific integrase	CWD84_RS07830
	AimR family lysis-lysogeny pheromone receptor	CWD84_RS07835
	Helix-turn-helix transcriptional regulator	CWD84_RS07840
	Helix-turn-helix transcriptional regulator	CWD84_RS07845
	Hypothetical protein	CWD84_RS07850
	Conserved phage C-terminal domain-containing protein	CWD84_RS07855
	ATP-binding protein	CWD84_RS07860
	Hypothetical protein	CWD84_RS07865
	XtrA/YqaO family protein	CWD84_RS07870
	Hypothetical protein	CWD84_RS07875
	Hypothetical protein	CWD84_RS07880
	Hypothetical protein	CWD84_RS07885
	DNA adenine methylase	CWD84_RS07890
	Hypothetical protein	CWD84_RS07895
	DUTP diphosphatase	CWD84_RS07900
	Hypothetical protein	CWD84_RS07905
	Hypothetical protein	CWD84_RS07910
	Hypothetical protein	CWD84_RS07915
	Hypothetical protein	CWD84_RS07920
	Hypothetical protein	CWD84_RS07925
	ArpU family transcriptional regulator	CWD84_RS07930
	Tyrosine-type recombinase/integrase	CWD84_RS07935
	Cell division protein FtsK	CWD84_RS07940
	Hypothetical protein	CWD84_RS07950
	Hypothetical protein	CWD84_RS07955
	Hypothetical protein	CWD84_RS07960
	Hypothetical protein	CWD84_RS07965
	HNH endonuclease	CWD84_RS07970
	Phage terminase small subunit P27 family	CWD84_RS07980
	Terminase large subunit	CWD84_RS07985
	Phage portal protein	CWD84_RS07990
	#VALUE!	CWD84_RS07995
	Phage major capsid protein	CWD84_RS08000
	Collagen-like protein	CWD84_RS08005
	Phage gp6-like head-tail connector protein	CWD84_RS08010
	Phage head closure protein	CWD84_RS08015
	HK97 gp10 family phage protein	CWD84_RS08020
	DUF3168 domain-containing protein	CWD84_RS08025
	Tail protein	CWD84_RS08030

Strain	Product	Gene locus
	Hypothetical protein	CWD84_RS08035
	Phage tail tape measure protein	CWD84_RS08040
	Phage tail family protein	CWD84_RS08045
	Phage tail protein	CWD84_RS08050
	Peptidase G2	CWD84_RS08055
	BppU family phage baseplate upper protein	CWD84_RS08060
	Hypothetical protein	CWD84_RS08065
	XkdX family protein	CWD84_RS08070
	Phage holin family protein	CWD84_RS08075
	N-acetylmuramoyl-L-alanine amidase	CWD84_RS08080
	Hypothetical protein	CWD84_RS08085
	Hypothetical protein	CWD84_RS08090
	Hypothetical protein	CWD84_RS08095
	Hypothetical protein	CWD84_RS08100
	Hypothetical protein	CWD84_RS08105
	Site-specific integrase	CWD84_RS08110
	AimR family lysis-lysogeny pheromone receptor	CWD84_RS08115
	Helix-turn-helix transcriptional regulator	CWD84_RS08120
	Helix-turn-helix transcriptional regulator	CWD84_RS08125
	Hypothetical protein	CWD84_RS08130
	Conserved phage C-terminal domain-containing protein	CWD84_RS08135
	ATP-binding protein	CWD84_RS08140
	Hypothetical protein	CWD84_RS08145
	XtrA/YqaO family protein	CWD84_RS08150
	Hypothetical protein	CWD84_RS08155
	Hypothetical protein	CWD84_RS08160
	Hypothetical protein	CWD84_RS08165
	DNA adenine methylase	CWD84_RS08170
	Hypothetical protein	CWD84_RS08175
	DUTP diphosphatase	CWD84_RS08180
	Hypothetical protein	CWD84_RS08185
	Hypothetical protein	CWD84_RS08190
	Hypothetical protein	CWD84_RS08195
	Hypothetical protein	CWD84_RS08200
	Hypothetical protein	CWD84_RS08205
	ArpU family transcriptional regulator	CWD84_RS08210
	Tyrosine-type recombinase/integrase	CWD84_RS08215
	Cell division protein FtsK	CWD84_RS08220
	Hypothetical protein	CWD84_RS08230
	Hypothetical protein	CWD84_RS08235
	Hypothetical protein	CWD84_RS08240
	Hypothetical protein	CWD84_RS08245
	HNH endonuclease	CWD84_RS08250
	Phage terminase small subunit P27 family	CWD84_RS08260

Strain	Product	Gene locus
	Terminase large subunit	CWD84_RS08265
	Phage portal protein	CWD84_RS08270
	HK97 family phage prohead protease	CWD84_RS08275
	Phage major capsid protein	CWD84_RS08280
	Collagen-like protein	CWD84_RS08285
	Phage gp6-like head-tail connector protein	CWD84_RS08290
	Phage head closure protein	CWD84_RS08295
	HK97 gp10 family phage protein	CWD84_RS08300
	DUF3168 domain-containing protein	CWD84_RS08305
	Tail protein	CWD84_RS08310
	Hypothetical protein	CWD84_RS08315
	Phage tail tape measure protein	CWD84_RS08320
	Phage tail family protein	CWD84_RS08325
	Phage tail protein	CWD84_RS08330
	Peptidase G2	CWD84_RS08335
	BppU family phage baseplate upper protein	CWD84_RS08340
	Hypothetical protein	CWD84_RS08345
	XkdX family protein	CWD84_RS08350
	Phage holin family protein	CWD84_RS08355
	N-acetylmuramoyl-L-alanine amidase	CWD84_RS08360
	Hypothetical protein	CWD84_RS08365
	Hypothetical protein	CWD84_RS08370
	Hypothetical protein	CWD84_RS08375
	Hypothetical protein	CWD84_RS08380
	Hypothetical protein	CWD84_RS08385
	Site-specific integrase	CWD84_RS08390
	AimR family lysis-lysogeny pheromone receptor	CWD84_RS08395
	Helix-turn-helix transcriptional regulator	CWD84_RS08400
	Helix-turn-helix transcriptional regulator	CWD84_RS08405
	Hypothetical protein	CWD84_RS08410
	Conserved phage C-terminal domain-containing protein	CWD84_RS08415
	ATP-binding protein	CWD84_RS08420
	Hypothetical protein	CWD84_RS08425
	XtrA/YqaO family protein	CWD84_RS08430
	Hypothetical protein	CWD84_RS08435
	Hypothetical protein	CWD84_RS08440
	Hypothetical protein	CWD84_RS08445
	DNA adenine methylase	CWD84_RS08450
	Hypothetical protein	CWD84_RS08455
	DUTP diphosphatase	CWD84_RS08460
	Hypothetical protein	CWD84_RS08465
	Hypothetical protein	CWD84_RS08470
	Hypothetical protein	CWD84_RS08475
	Hypothetical protein	CWD84_RS08480

Strain	Product	Gene locus
	Hypothetical protein	CWD84_RS08485
	ArpU family transcriptional regulator	CWD84_RS08490
	Tyrosine-type recombinase/integrase	CWD84_RS08495
	Cell division protein FtsK	CWD84_RS08500
	Hypothetical protein	CWD84_RS08510
	Hypothetical protein	CWD84_RS08515
	Hypothetical protein	CWD84_RS08520
	Hypothetical protein	CWD84_RS08525
	HNH endonuclease	CWD84_RS08530
	Phage terminase small subunit P27 family	CWD84_RS08540
	Terminase large subunit	CWD84_RS08545
	Phage portal protein	CWD84_RS08550
	HK97 family phage prohead protease	CWD84_RS08555
	Phage major capsid protein	CWD84_RS08560
	Collagen-like protein	CWD84_RS08565
	Phage gp6-like head-tail connector protein	CWD84_RS08570
	Phage head closure protein	CWD84_RS08575
	HK97 gp10 family phage protein	CWD84_RS08580
	DUF3168 domain-containing protein	CWD84_RS08585
	Tail protein	CWD84_RS08590
	Hypothetical protein	CWD84_RS08595
	Phage tail tape measure protein	CWD84_RS08600
	Phage tail family protein	CWD84_RS08605
	Phage tail protein	CWD84_RS08610
	Peptidase G2	CWD84_RS08615
	BppU family phage baseplate upper protein	CWD84_RS08620
	Hypothetical protein	CWD84_RS08625
	XkdX family protein	CWD84_RS08630
	Phage holin family protein	CWD84_RS08635
	N-acetylmuramoyl-L-alanine amidase	CWD84_RS08640
	Hypothetical protein	CWD84_RS08645
	Hypothetical protein	CWD84_RS08650
	Hypothetical protein	CWD84_RS08655
	Hypothetical protein	CWD84_RS08660
	Hypothetical protein	CWD84_RS08665
	XkdX family protein	CWD84_RS12330
	IS1182 family transposase	CWD84_RS16895
	IS3 family transposase	CWD84_RS17860
	IS1182 family transposase	CWD84_RS20370
	BH0509 family protein	CWD84_RS22070
	BH0509 family protein	CWD84_RS22075
	BH0509 family protein	CWD84_RS22080
	BH0509 family protein	CWD84_RS22085
	Hypothetical protein	CWD84_RS22235

Strain	Product	Gene locus
	Hypothetical protein	CWD84_RS22240
	Hypothetical protein	CWD84_RS22245
	Hypothetical protein	CWD84_RS22250
	Hypothetical protein	CWD84_RS22260
	Hypothetical protein	CWD84_RS22265
	Hypothetical protein	CWD84_RS22270
	Hypothetical protein	CWD84_RS22275
	Hypothetical protein	CWD84_RS22285
	Hypothetical protein	CWD84_RS22290
	Hypothetical protein	CWD84_RS22295
	Hypothetical protein	CWD84_RS22300
	Hypothetical protein	CWD84_RS22310
	Hypothetical protein	CWD84_RS22315
	Hypothetical protein	CWD84_RS22320
	Hypothetical protein	CWD84_RS22325
	Hypothetical protein	CWD84_RS00195
	Hypothetical protein	CWD84_RS00210
	Hypothetical protein	CWD84_RS00265
	Restriction endonuclease	CWD84_RS00285
	Hypothetical protein	CWD84_RS00295
	ATP-binding cassette domain-containing protein	CWD84_RS00300
	Hypothetical protein	CWD84_RS00305
	Hypothetical protein	CWD84_RS00310
	Hypothetical protein	CWD84_RS00315
	Hypothetical protein	CWD84_RS00320
	Hypothetical protein	CWD84_RS00325
	EamA family transporter	CWD84_RS00340
	PLP-dependent aminotransferase family protein	CWD84_RS00345
	Protein kinase	CWD84_RS00640
	Hypothetical protein	CWD84_RS00645
	Hypothetical protein	CWD84_RS00650
	Replication ATP-dependent helicase	CWD84_RS00655
	2-keto-myo-inositol isomerase	CWD84_RS00845
	FRG domain-containing protein	CWD84_RS00940
	DUF262 domain-containing protein	CWD84_RS00945
	Hypothetical protein	CWD84_RS00980
	Hypothetical protein	CWD84_RS00985
	Hypothetical protein	CWD84_RS00990
	Hypothetical protein	CWD84_RS00995
	Hypothetical protein	CWD84_RS01000
	Hypothetical protein	CWD84_RS01005
	DNA-binding protein	CWD84_RS01015
	Hypothetical protein	CWD84_RS01030
	DUF2716 domain-containing protein	CWD84_RS01035

Strain	Product	Gene locus
	SMI1/KNR4 family protein	CWD84_RS01040
	Hypothetical protein	CWD84_RS01045
	Sigma 54-interacting transcriptional regulator	CWD84_RS01260
	Agmatinase	CWD84_RS01265
	Sodium:solute symporter	CWD84_RS01270
	Aldehyde dehydrogenase family protein	CWD84_RS01275
	Cytosine permease	CWD84_RS01280
	D-alanyl-lipoteichoic acid biosynthesis protein DltD	CWD84_RS01290
	FTR1 family protein	CWD84_RS01390
	EfeM/EfeO family lipoprotein	CWD84_RS01395
	Cupin domain-containing protein	CWD84_RS01665
	Dihydroanticapsin 7-dehydrogenase	CWD84_RS01670
	ATP-grasp domain-containing protein	CWD84_RS01675
	MFS transporter	CWD84_RS01680
	Pyridoxal phosphate-dependent aminotransferase	CWD84_RS01685
	Hypothetical protein	CWD84_RS01720
	Hypothetical protein	CWD84_RS01725
	Hypothetical protein	CWD84_RS01730
	Hypothetical protein	CWD84_RS01735
	ABC transporter permease	CWD84_RS01740
	ATP-binding cassette domain-containing protein	CWD84_RS01745
	YwiC-like family protein	CWD84_RS01860
	ABC transporter ATP-binding protein	CWD84_RS01900
	DUF5082 family protein	CWD84_RS02380
	YwqI/YxiC family protein	CWD84_RS02385
	Transposase	CWD84_RS02390
	SUKH-4 family immunity protein	CWD84_RS02395
	Hypothetical protein	CWD84_RS02400
	Hypothetical protein	CWD84_RS02405
	Glycosyltransferase family 2 protein	CWD84_RS02625
	Hypothetical protein	CWD84_RS02660
	SpoIID/LytB domain-containing protein	CWD84_RS02665
	N-acetylmuramoyl-L-alanine amidase	CWD84_RS02670
	Hypothetical protein	CWD84_RS03135
	Hypothetical protein	CWD84_RS03180
	Lipase	CWD84_RS03195
	DUF1433 domain-containing protein	CWD84_RS03200
	DUF1433 domain-containing protein	CWD84_RS03215
	DUF1433 domain-containing protein	CWD84_RS03225
	DUF1433 domain-containing protein	CWD84_RS03230
	Hypothetical protein	CWD84_RS03425
	IS1182 family transposase	CWD84_RS03495
	Amidohydrolase family protein	CWD84_RS03705
	Carboxymuconolactone decarboxylase family protein	CWD84_RS03710

Strain	Product	Gene locus
	Helix-turn-helix domain-containing protein	CWD84_RS03720
	DUF1657 domain-containing protein	CWD84_RS04255
	Hypothetical protein	CWD84_RS04260
	Stage V sporulation protein AC	CWD84_RS04265
	Stage V sporulation protein AD	CWD84_RS04270
	Stage V sporulation protein AE	CWD84_RS04275
	DUF1657 domain-containing protein	CWD84_RS04280
	DUF421 domain-containing protein	CWD84_RS04285
	SdpA family antimicrobial peptide system protein	CWD84_RS04335
	Hypothetical protein	CWD84_RS04340
	Sporulation delaying protein family toxin	CWD84_RS04345
	Tyrosine-type recombinase/integrase	CWD84_RS04615
	Helix-turn-helix domain-containing protein	CWD84_RS04620
	Site-specific integrase	CWD84_RS04625
	Helix-turn-helix transcriptional regulator	CWD84_RS04630
	Hypothetical protein	CWD84_RS04635
	Helix-turn-helix transcriptional regulator	CWD84_RS04640
	AimR family lysis-lysogeny pheromone receptor	CWD84_RS04645
	Hypothetical protein	CWD84_RS04650
	Hypothetical protein	CWD84_RS04655
	Helix-turn-helix transcriptional regulator	CWD84_RS04660
	Hypothetical protein	CWD84_RS04665
	Hypothetical protein	CWD84_RS04670
	Hypothetical protein	CWD84_RS04680
	Hypothetical protein	CWD84_RS04685
	Hypothetical protein	CWD84_RS04690
	Hypothetical protein	CWD84_RS04695
	Hypothetical protein	CWD84_RS04700
	Phage tail tape measure protein	CWD84_RS04705
	SHOCT domain-containing protein	CWD84_RS04710
	WXG100 family type VII secretion target	CWD84_RS04870
	EsaB/YukD family protein	CWD84_RS04875
	Type VII secretion protein EssB	CWD84_RS04880
	Type VII secretion protein EssC	CWD84_RS04885
	Type VII secretion protein EsaA	CWD84_RS04890
	Type VII secretion protein EssA	CWD84_RS04895
	YueI family protein	CWD84_RS04930
	Competence pheromone ComX	CWD84_RS04970
	Tetratricopeptide repeat protein	CWD84_RS05270
	Glycerophosphodiester phosphodiesterase	CWD84_RS05630
	Hypothetical protein	CWD84_RS05920
	N-acetylmuramoyl-L-alanine amidase	CWD84_RS05930
	CGNR zinc finger domain-containing protein	CWD84_RS05960
	HPP family protein	CWD84_RS05965

Strain	Product	Gene locus
	NO-inducible flavohemoprotein	CWD84_RS06105
	Hypothetical protein	CWD84_RS06115
	HNH endonuclease	CWD84_RS06135
	Aldehyde dehydrogenase	CWD84_RS06145
	Hypothetical protein	CWD84_RS06295
	Sugar ABC transporter permease	CWD84_RS06545
	TIGR04141 family sporadically distributed protein	CWD84_RS06770
	Hypothetical protein	CWD84_RS06775
	AimR family lysis-lysogeny pheromone receptor	CWD84_RS07275
	Helix-turn-helix transcriptional regulator	CWD84_RS07280
	Helix-turn-helix transcriptional regulator	CWD84_RS07285
	Hypothetical protein	CWD84_RS07290
	Conserved phage C-terminal domain-containing protein	CWD84_RS07295
	ATP-binding protein	CWD84_RS07300
	Hypothetical protein	CWD84_RS07305
	XtrA/YqaO family protein	CWD84_RS07310
	Hypothetical protein	CWD84_RS07315
	Hypothetical protein	CWD84_RS07320
	Hypothetical protein	CWD84_RS07325
	DNA adenine methylase	CWD84_RS07330
	Hypothetical protein	CWD84_RS07335
	Hypothetical protein	CWD84_RS07345
	Hypothetical protein	CWD84_RS07360
	Hypothetical protein	CWD84_RS07365
	Tyrosine-type recombinase/integrase	CWD84_RS07375
	Hypothetical protein	CWD84_RS07390
	Hypothetical protein	CWD84_RS07400
	Hypothetical protein	CWD84_RS07405
	Peptidase G2	CWD84_RS07495
	BppU family phage baseplate upper protein	CWD84_RS07500
	N-acetylmuramoyl-L-alanine amidase	CWD84_RS07520
	Hypothetical protein	CWD84_RS07525
	Hypothetical protein	CWD84_RS07530
	Hypothetical protein	CWD84_RS07535
	Hypothetical protein	CWD84_RS07540
	Hypothetical protein	CWD84_RS07545
	AraC family transcriptional regulator	CWD84_RS08825
	Hypothetical protein	CWD84_RS08860
	Site-specific integrase	CWD84_RS08875
	Antiterminator LoaP	CWD84_RS09900
	D-fructose-6-phosphate amidotransferase	CWD84_RS09915
	Acyl carrier protein	CWD84_RS09920
	Long-chain fatty acid--CoA ligase	CWD84_RS09925
	SDR family oxidoreductase	CWD84_RS09930

Strain	Product	Gene locus
	SDR family NAD(P)-dependent oxidoreductase	CWD84_RS09935
	KR domain-containing protein	CWD84_RS09940
	SDR family NAD(P)-dependent oxidoreductase	CWD84_RS09945
	SDR family NAD(P)-dependent oxidoreductase	CWD84_RS09950
	SDR family NAD(P)-dependent oxidoreductase	CWD84_RS09955
	SDR family NAD(P)-dependent oxidoreductase	CWD84_RS09960
	Zinc-binding dehydrogenase	CWD84_RS09965
	Cytochrome P450	CWD84_RS09970
	Hydroxymethylglutaryl-CoA synthase family protein	CWD84_RS09975
	Enoyl-CoA hydratase/isomerase family protein	CWD84_RS09980
	Hypothetical protein	CWD84_RS10125
	Ribonuclease YeeF family protein	CWD84_RS11090
	SMI1/KNR4 family protein	CWD84_RS11100
	Putative holin-like toxin	CWD84_RS11105
	Hypothetical protein	CWD84_RS11120
	Ribonuclease YeeF family protein	CWD84_RS11595
	SMI1/KNR4 family protein	CWD84_RS11600
	Molybdopterin oxidoreductase family protein	CWD84_RS11625
	Non-ribosomal peptide synthetase	CWD84_RS11765
	Non-ribosomal peptide synthetase	CWD84_RS11770
	Non-ribosomal peptide synthetase	CWD84_RS11775
	Non-ribosomal peptide synthetase	CWD84_RS11780
	DUF1453 family protein	CWD84_RS12055
	Hypothetical protein	CWD84_RS12110
	Hypothetical protein	CWD84_RS12275
	HNH endonuclease	CWD84_RS12280
	SMI1/KNR4 family protein	CWD84_RS12285
	Peptidoglycan-binding protein	CWD84_RS12290
	N-acetylmuramoyl-L-alanine amidase	CWD84_RS12315
	Phage holin	CWD84_RS12320
	Hypothetical protein	CWD84_RS12325
	Hypothetical protein	CWD84_RS12335
	Phage baseplate upper protein	CWD84_RS12340
	Peptidase G2	CWD84_RS12345
	Phage tail protein	CWD84_RS12350
	Phage tail family protein	CWD84_RS12355
	Phage tail tape measure protein	CWD84_RS12360
	Hypothetical protein	CWD84_RS12365
	Hypothetical protein	CWD84_RS12370
	UDP-N-acetylmuramoylalanine--D-glutamate ligase	CWD84_RS12375
	DUF3168 domain-containing protein	CWD84_RS12380
	HK97 gp10 family phage protein	CWD84_RS12385
	Phage head closure protein	CWD84_RS12390
	Phage gp6-like head-tail connector protein	CWD84_RS12395

Strain	Product	Gene locus
	Collagen-like protein	CWD84_RS12400
	Phage major capsid protein	CWD84_RS12405
	HK97 family phage prohead protease	CWD84_RS12410
	Phage portal protein	CWD84_RS12415
	Hypothetical protein	CWD84_RS12420
	Terminase large subunit	CWD84_RS12425
	Phage terminase small subunit P27 family	CWD84_RS12430
	Hypothetical protein	CWD84_RS12440
	Hypothetical protein	CWD84_RS12445
	DNA-binding protein	CWD84_RS12505
	Phage antirepressor KilAC domain-containing protein	CWD84_RS12510
	Helix-turn-helix transcriptional regulator	CWD84_RS12515
	Helix-turn-helix transcriptional regulator	CWD84_RS12520
	ImmA/IrrE family metallo-endopeptidase	CWD84_RS12525
	Site-specific integrase	CWD84_RS12530
	Ribonuclease YeeF family protein	CWD84_RS12750
	Hypothetical protein	CWD84_RS12755
	Hypothetical protein	CWD84_RS12760
	Hypothetical protein	CWD84_RS12765
	Trypsin-like peptidase domain-containing protein	CWD84_RS12770
	L-serine ammonia-lyase, iron-sulfur-dependent, subunit alpha	CWD84_RS13380
	Serine hydrolase	CWD84_RS14035
	Alpha/beta fold hydrolase	CWD84_RS14040
	SDR family NAD(P)-dependent oxidoreductase	CWD84_RS14045
	SDR family NAD(P)-dependent oxidoreductase	CWD84_RS14050
	SDR family NAD(P)-dependent oxidoreductase	CWD84_RS14055
	SDR family NAD(P)-dependent oxidoreductase	CWD84_RS14060
	SDR family NAD(P)-dependent oxidoreductase	CWD84_RS14065
	SDR family NAD(P)-dependent oxidoreductase	CWD84_RS14070
	ACP S-malonyltransferase	CWD84_RS14075
	PAS domain-containing protein	CWD84_RS14370
	Hypothetical protein	CWD84_RS14385
	Glucose 1-dehydrogenase	CWD84_RS14480
	Winged helix-turn-helix transcriptional regulator	CWD84_RS14485
	XkdW family protein	CWD84_RS14950
	Hypothetical protein	CWD84_RS15090
	Cupin domain-containing protein	CWD84_RS15165
	Hypothetical protein	CWD84_RS15235
	Galactose oxidase	CWD84_RS15240
	Hypothetical protein	CWD84_RS15315
	Hypothetical protein	CWD84_RS15320
	Hypothetical protein	CWD84_RS15325
	Hypothetical protein	CWD84_RS15330
	Hypothetical protein	CWD84_RS15335

Strain	Product	Gene locus
	Hypothetical protein	CWD84_RS15370
	Hypothetical protein	CWD84_RS15375
	YjdJ family protein	CWD84_RS15420
	Hypothetical protein	CWD84_RS15425
	Hypothetical protein	CWD84_RS15435
	Abi family protein	CWD84_RS15445
	N-acetylmuramoyl-L-alanine amidase	CWD84_RS15450
	Phage holin family protein	CWD84_RS15455
	Hypothetical protein	CWD84_RS15460
	XkdX family protein	CWD84_RS15465
	Hypothetical protein	CWD84_RS15470
	Phage tail protein	CWD84_RS15475
	Phage tail family protein	CWD84_RS15480
	Hypothetical protein	CWD84_RS15485
	Hypothetical protein	CWD84_RS15490
	Hypothetical protein	CWD84_RS15495
	Fibronectin type III domain-containing protein	CWD84_RS15500
	Fibronectin type III domain-containing protein	CWD84_RS15505
	Phage major tail protein, TP901-1 family	CWD84_RS15510
	DUF3168 domain-containing protein	CWD84_RS15515
	HK97 gp10 family phage protein	CWD84_RS15520
	Phage head closure protein	CWD84_RS15525
	Phage head-tail connector protein	CWD84_RS15530
	Hypothetical protein	CWD84_RS15535
	Hypothetical protein	CWD84_RS15540
	Phage major capsid protein	CWD84_RS15545
	DUF4355 domain-containing protein	CWD84_RS15550
	Hypothetical protein	CWD84_RS15555
	Phage head morphogenesis protein	CWD84_RS15560
	Phage portal protein	CWD84_RS15565
	PBSX family phage terminase large subunit	CWD84_RS15570
	Hypothetical protein	CWD84_RS15575
	Hypothetical protein	CWD84_RS15580
	Hypothetical protein	CWD84_RS15585
	Sigma-70 family RNA polymerase sigma factor	CWD84_RS15600
	Hypothetical protein	CWD84_RS15605
	Hypothetical protein	CWD84_RS15615
	Hypothetical protein	CWD84_RS15625
	Hypothetical protein	CWD84_RS15640
	Hypothetical protein	CWD84_RS15645
	DUF1064 domain-containing protein	CWD84_RS15660
	Hypothetical protein	CWD84_RS15665
	Hypothetical protein	CWD84_RS15670
	ATP-binding protein	CWD84_RS15675

Strain	Product	Gene locus
	Rha family transcriptional regulator	CWD84_RS15725
	Helix-turn-helix domain-containing protein	CWD84_RS15730
	Helix-turn-helix transcriptional regulator	CWD84_RS15735
	Helix-turn-helix transcriptional regulator	CWD84_RS15740
	ImmA/IrrE family metallo-endopeptidase	CWD84_RS15755
	Site-specific integrase	CWD84_RS15760
	5-nucleotidase C-terminal domain-containing protein	CWD84_RS17045
	Hypothetical protein	CWD84_RS17315
	Hypothetical protein	CWD84_RS17320
	Hypothetical protein	CWD84_RS17325
	Hypothetical protein	CWD84_RS17330
	Hypothetical protein	CWD84_RS17500
	DUF2977 domain-containing protein	CWD84_RS17525
	Hypothetical protein	CWD84_RS17540
	Hypothetical protein	CWD84_RS17620
	Hypothetical protein	CWD84_RS17625
	Hypothetical protein	CWD84_RS17630
	Hypothetical protein	CWD84_RS17635
	Type II toxin-antitoxin system HicA family toxin	CWD84_RS17640
	Type II toxin-antitoxin system HicB family antitoxin	CWD84_RS17645
	Sigma-70 family RNA polymerase sigma factor	CWD84_RS17650
	XtrA/YqaO family protein	CWD84_RS17655
	Replicative DNA helicase	CWD84_RS17660
	Hypothetical protein	CWD84_RS17665
	Hypothetical protein	CWD84_RS17670
	Hypothetical protein	CWD84_RS17675
	Hypothetical protein	CWD84_RS17680
	Rha family transcriptional regulator	CWD84_RS17685
	Hypothetical protein	CWD84_RS17690
	Hypothetical protein	CWD84_RS17695
	Hypothetical protein	CWD84_RS17700
	Helix-turn-helix transcriptional regulator	CWD84_RS17705
	Helix-turn-helix transcriptional regulator	CWD84_RS17710
	ImmA/IrrE family metallo-endopeptidase	CWD84_RS17715
	Site-specific integrase	CWD84_RS17720
	Phosphoenolpyruvate synthase	CWD84_RS17835
	HAD family phosphatase	CWD84_RS18245
	Cytochrome P450	CWD84_RS18250
	NCS2 family permease	CWD84_RS18255
	GNAT family N-acetyltransferase	CWD84_RS18385
	Ribonuclease YeeF family protein	CWD84_RS18415
	Antitoxin YezG family protein	CWD84_RS18420
	TIGR01741 family protein	CWD84_RS18425
	DUF4062 domain-containing protein	CWD84_RS18435

Strain	Product	Gene locus
	Protein kinase	CWD84_RS18440
	ABC transporter substrate-binding protein	CWD84_RS18675
	TauD/TfdA family dioxygenase	CWD84_RS18685
	ATP-grasp domain-containing protein	CWD84_RS18690
	MFS transporter	CWD84_RS18695
	ATP-grasp domain-containing protein	CWD84_RS18700
	Hypothetical protein	CWD84_RS18705
	Hypothetical protein	CWD84_RS18810
	tRNA (adenosine(37)-N6)-threonylcarbamoyltransferase complex dimerization subunit type 1 TsaB	CWD84_RS18875
	Sugar O-acetyltransferase	CWD84_RS19095
	ROK family protein	CWD84_RS19100
	MFS transporter	CWD84_RS19105
	NAD(P)-dependent alcohol dehydrogenase	CWD84_RS19180
	VTT domain-containing protein	CWD84_RS19210
	Hypothetical protein	CWD84_RS19275
	Aminotransferase class I/II-fold pyridoxal phosphate-dependent enzyme	CWD84_RS19280
	TSUP family transporter	CWD84_RS19295
	DHA2 family efflux MFS transporter permease subunit	CWD84_RS19305
	MFS transporter	CWD84_RS19310
	Molybdopterin-dependent oxidoreductase	CWD84_RS19315
	LysR family transcriptional regulator	CWD84_RS19320
	Molecular chaperone TorD family protein	CWD84_RS19335
	MaoC family dehydratase	CWD84_RS19355
	Tunicamycin resistance ATP-binding TmrB	CWD84_RS19430
	Cystatin-like fold lipoprotein	CWD84_RS19435
	Manganese catalase family protein	CWD84_RS19650
	Aldo/keto reductase family oxidoreductase	CWD84_RS19835
	Amino acid ABC transporter substrate-binding protein	CWD84_RS20070
	Right-handed parallel beta-helix repeat-containing protein	CWD84_RS20105
	Antimicrobial peptide LCI	CWD84_RS20305
	MFS transporter	CWD84_RS20455
	Ribonuclease YeeF family protein	CWD84_RS20670
	Hypothetical protein	CWD84_RS20675
	DUF1906 domain-containing protein	CWD84_RS20680
	DUF2651 family protein	CWD84_RS20715
	Hypothetical protein	CWD84_RS20720
	Hypothetical protein	CWD84_RS20725
	Sensor histidine kinase	CWD84_RS20780
	MarR family transcriptional regulator	CWD84_RS20795
	CidA/LrgA family holin-like protein	CWD84_RS20800
	LrgB family protein	CWD84_RS20805
	AAA family ATPase	CWD84_RS20825
	AAA family ATPase	CWD84_RS20830
	AraC family transcriptional regulator	CWD84_RS20925

Strain	Product	Gene locus
	Hypothetical protein	CWD84_RS21980*
	Hypothetical protein	CWD84_RS21985*
	Hypothetical protein	CWD84_RS21990*
	Replication initiation protein	CWD84_RS21995*
	Hypothetical protein	CWD84_RS22000*
	Hypothetical protein	CWD84_RS22005*
	Hypothetical protein	CWD84_RS22010*
	Hypothetical protein	CWD84_RS22015*
	Hypothetical protein	CWD84_RS22020*
	AAA family ATPase	CWD84_RS22025*
	Hypothetical protein	CWD84_RS22030*
	Hypothetical protein	CWD84_RS22035*
	Hypothetical protein	CWD84_RS22040*
	Hypothetical protein	CWD84_RS22045*
	Fibronectin type III domain-containing protein	CWD84_RS22050*
	Hypothetical protein	CWD84_RS22055*
	BH0509 family protein	CWD84_RS22065
	Hypothetical protein	CWD84_RS22165
	Hypothetical protein	CWD84_RS22170
	Hypothetical protein	CWD84_RS22175
	Hypothetical protein	CWD84_RS22180
	Hypothetical protein	CWD84_RS22190
	Hypothetical protein	CWD84_RS22195
	Type VI secretion system contractile sheath protein Tss	CWD84_RS22205
	Hypothetical protein	CWD84_RS22210
	Hypothetical protein	CWD84_RS22215
	Hypothetical protein	CWD84_RS22220
	Hypothetical protein	CWD84_RS22225
	Hypothetical protein	CWD84_RS22365
	Hypothetical protein	CWD84_RS22385
	Hypothetical protein	CWD84_RS22395
	Hypothetical protein	CWD84_RS22425
	Hypothetical protein	CWD84_RS22435
	Hypothetical protein	CWD84_RS22440
	Hypothetical protein	CWD84_RS22455
	Hypothetical protein	CWD84_RS22460*
	Hypothetical protein	CWD84_RS22465
	Iron-containing redox enzyme family protein	CWD84_RS22470

*Genes located in a plasmid.

Table S2. List of the genes for probiotic properties.

Gene locus	EC. No.	Product	COG
Survivability			
JD965_RS18870	3.5.1.24	Choloylglycine hydrolase	G
Adhesion			
JD965_RS08405	-	Fibronectin-binding protein	K
Biofilm formation			
JD965_RS00290	-	Protein Veg	S
JD965_RS16825	2.-.-.-	Putative pyruvyl transferase EpsO	M
JD965_RS16830	2.6.1.-	Putative pyridoxal phosphate-dependent aminotransferase EpsN	E
JD965_RS16835	2.3.1.-	Putative acetyltransferase EpsM	S
JD965_RS16840	2.-.-.-	Uncharacterized sugar transferase EpsL	M
JD965_RS16850	2.4.-.-	Chondroitin synthase	M
JD965_RS16855	2.-.-.-	Putative pyruvyl transferase EpsI	M
JD965_RS16860	2.4.-.-	Putative glycosyltransferase EpsH	M
JD965_RS16870	2.4.-.-	Putative glycosyltransferase EpsF	M
JD965_RS16875	2.4.-.-	Putative glycosyltransferase EpsE	M
JD965_RS16880	2.4.-.-	Putative glycosyltransferase EpsD	M
JD965_RS16890	2.7.10.2	Non-specific protein-tyrosine kinase	D
JD965_RS16900	-	HTH-type transcriptional regulator SlrR	K
JD965_RS16905	3.1.1.-	Probable secreted lipase	I
JD965_RS17730	2.7.10.2	Non-specific protein-tyrosine kinase	D
JD965_RS18685	-	Transcriptional regulator SlrA	
JD965_RS19630	2.4.-.-	N,N'-diacetylbacillosaminyl-diphospho-undecaprenol alpha-1,3-N-acetylgalactosaminyltransferase	M
JD965_RS19645	2.4.-.-	Amylovoran biosynthesis glycosyltransferase AmsK	M
Flagellum			
JD965_RS04275	-	hypothetical protein	
JD965_RS04285	-	Collagen alpha-6(IV) chain	S
JD965_RS07180	-	hypothetical protein	S
JD965_RS07195	3.6.3.17	Monosaccharide-transporting ATPase	S
JD965_RS07425	-	Motility protein	N
JD965_RS08625	-	uncharacterized protein	S
JD965_RS08675	-	Flagellar basal body rod protein FlgB	N
JD965_RS08680	-	Flagellar basal-body rod protein FlgC	N
JD965_RS08685	-	Flagellar hook-basal body complex protein FliE	N
JD965_RS08690	-	Flagellar M-ring protein	N
JD965_RS08700	-	Probable flagellar assembly protein FliH	N
JD965_RS08705	3.6.3.14	H(+)-transporting two-sector ATPase	NU
JD965_RS08710	-	Flagellar FliJ protein	N
JD965_RS08715	-	FlaA locus 22.9 kDa protein	S

Gene locus	EC. No.	Product	COG
JD965_RS08720	-	Probable flagellar hook-length control protein	N
JD965_RS08725	-	FlaA locus uncharacterized protein YlxG	N
JD965_RS08730	-	Flagellar hook protein FlgE	N
JD965_RS08740	-	Flagellar protein FliL	N
JD965_RS08760	-	Flagellar biosynthetic protein FliZ	N
JD965_RS08765	-	Flagellar biosynthetic protein FliP	N
JD965_RS08770	-	Flagellar biosynthetic protein FliQ	N
JD965_RS08775	-	Flagellar biosynthetic protein FliR	N
JD965_RS08780	-	Flagellar biosynthetic protein FlhB	N
JD965_RS08785	-	Flagellar biosynthesis protein FlhA	N
JD965_RS08790	-	Flagellar biosynthesis protein FlhF	N
JD965_RS08795	-	Flagellum site-determining protein YlxH	D
JD965_RS08825	-	RNA polymerase sigma-D factor	K
JD965_RS09195	-	Polyketide synthase	Q
JD965_RS14480	-	Uncharacterized 24.6 kDa protein in ccpA 3'region	N
JD965_RS14485	-	uncharacterized protein	N
JD965_RS15125	3.2.1.17	Lysozyme	NU
JD965_RS16815	-	RNA polymerase sigma-54 factor	K
JD965_RS17305	-	Flagellar protein FliS	N
JD965_RS17310	-	Flagellar hook-associated protein	N
JD965_RS17315	-	Flagellin	N
JD965_RS17325	-	Flagellar assembly factor FliW	S
JD965_RS17335	-	Flagellar hook-associated protein	N
JD965_RS17340	-	Flagellar hook-associated protein	N
JD965_RS17350	-	Negative regulator of flagellin synthesis	K
JD965_RS17355	-	uncharacterized protein	S
JD965_RS17380	-	Transcriptional regulatory protein DegU	T
JD965_RS17385	2.7.13.3	Histidine kinase	T
JD965_RS17795	-	Flagellar hook-basal body complex protein FlhP	N
JD965_RS17800	-	Flagellar hook-basal body complex protein FlhO	N
JD965_RS18255	-	uncharacterized protein	S
Bacitracin operon			
JD965_RS14710		Bacitracin export permease protein BceB	V
JD965_RS14715		Lipoprotein-releasing system ATP-binding protein LolD	V
JD965_RS14720	2.7.13.3	Histidine kinase	T
JD965_RS14725		Regulatory protein VirG	T
JD965_RS16175		Spore germination protein A3	S
JD965_RS16180		Chemotaxis response regulator protein-glutamate methylesterase of group 1 operon	T
JD965_RS16185	2.7.13.3	Histidine kinase	T
JD965_RS16190		Protein LiaF	S

Gene locus	EC. No.	Product	COG
JD965_RS16195		Protein LiaG	S
JD965_RS16200		Protein LiaH	KT
JD965_RS16205		Protein LiaI	
JD965_RS16210		Uncharacterized MFS-type transporter YvqJ	G
JD965_RS19160		ABC transporter permease protein YxdM	V
JD965_RS19165	3.6.3.-	Energy-coupling factor transporter ATP-binding protein EcfA1	V
JD965_RS19170	2.7.13.3	Histidine kinase	T
JD965_RS19175		Response regulator ArlR	T
Mesentericin operon			
JD965_RS19305		Hypothetical protein	
JD965_RS19310		Hypothetical protein	
JD965_RS19315		Hypothetical protein	
JD965_RS19320		Mesentericin-Y105 transport/processing ATP-binding protein MesD	V
JD965_RS19325		Mesentericin Y105 secretion protein MesE	U
Spore cortex			
JD965_RS00230	-	Stage 0 sporulation protein YaaT	S
JD965_RS00285	-	Sporulation-specific protease YabG	S
JD965_RS00350	-	Stage V sporulation protein	K
JD965_RS01250	-	Sigma-G-dependent sporulation-specific SASP protein	S
JD965_RS02395	-	Sporulation protein YdcC	M
JD965_RS02745	-	Putative sporulation hydrolase CotR	S
JD965_RS02840	-	Protease synthase and sporulation protein PAI	K
JD965_RS05295	-	Stage V sporulation protein	S
JD965_RS05550	-	Sporulation protein YhaL	S
JD965_RS06415	-	Sporulation-specific transcription factor SpoVIF	S
JD965_RS07020	-	Stage II sporulation protein SB	
JD965_RS07025	-	Stage II sporulation protein SA	
JD965_RS07245	3.4.-.-	Putative sporulation-specific glycosylase YdhD	G
JD965_RS07500	-	Sporulation protein YkvU	M
JD965_RS07505	-	Sporulation thiol-disulfide oxidoreductase	O
JD965_RS07710	-	Sporulation protein cse15	A
JD965_RS07865	-	UPF0752 sporulation protein YjcZ	S
JD965_RS08115	-	Sporulation integral membrane protein YlbJ	S
JD965_RS08185	-	Stage V sporulation protein D	M
JD965_RS08205	-	Stage V sporulation protein	D
JD965_RS08240	3.4.23.-	Sporulation sigma-E factor-processing peptidase	S
JD965_RS09080	-	Stage V sporulation protein	S
JD965_RS09205	-	Sporulation-specific extracellular nuclease	S
JD965_RS09305	-	Stage V sporulation protein	O
JD965_RS09530	-	Sporulation inhibitor of replication protein SirA	S

Gene locus	EC. No.	Product	COG
JD965_RS09855	-	Stage V sporulation protein AEB	S
JD965_RS09860	-	Stage V sporulation protein AD	S
JD965_RS09865	-	Stage V sporulation protein AC	S
JD965_RS11230	-	Stage IV sporulation protein	S
JD965_RS11290	-	Sporulation protein YpeB	S
JD965_RS11500	-	Stage V sporulation protein AF	S
JD965_RS11505	-	Stage V sporulation protein AE	S
JD965_RS11510	-	Stage V sporulation protein AEB	S
JD965_RS11515	-	Stage V sporulation protein AD	S
JD965_RS11520	-	Stage V sporulation protein AC	S
JD965_RS11525	-	Stage V sporulation protein	S
JD965_RS11530	-	Stage V sporulation protein AA	S
JD965_RS11580	-	Stage II sporulation protein	S
JD965_RS11995	-	Stage 0 sporulation protein	T
JD965_RS12065	-	Stage III sporulation protein AH	S
JD965_RS12070	-	Stage III sporulation protein AG	S
JD965_RS12075	-	Stage III sporulation protein AF	S
JD965_RS12080	-	Stage III sporulation protein AE	S
JD965_RS12085	-	Stage III sporulation protein AD	S
JD965_RS12090	-	Stage III sporulation protein AC	S
JD965_RS12095	-	Stage III sporulation protein	S
JD965_RS12100	-	Stage III sporulation protein AA	S
JD965_RS12640	-	Stage II sporulation protein	S
JD965_RS13175	-	Stage V sporulation protein	M
JD965_RS13230	-	Sporulation cortex protein CoxA	
JD965_RS13280	2.7.-.-	Sporulation initiation phosphotransferase	S
JD965_RS13300	3.4.24.-	Stage IV sporulation protein FB	S
JD965_RS13305	-	Stage IV sporulation protein FA	M
JD965_RS13595	-	Stage II sporulation protein	
JD965_RS13625	-	Stage VI sporulation protein D	S
JD965_RS14225	-	Sporulation membrane protein YtrH	S
JD965_RS14230	-	Sporulation membrane protein YtrI	S
JD965_RS14645	-	Sporulation protein cse60	S
JD965_RS15765	-	Sporulation protein YunB	S
JD965_RS17030	-	Putative sporulation transcription regulator WhiA	K
JD965_RS17810	-	Stage III sporulation protein	K
JD965_RS17865	-	Stage II sporulation protein	M
JD965_RS17960	-	Stage II sporulation protein D	D
JD965_RS18070	-	Stage II sporulation protein	S
JD965_RS18150	-	Sporulation initiation phosphotransferase	T

Gene locus	EC. No.	Product	COG
JD965_RS20050	-	Stage 0 sporulation protein J	K
Spore coat			
JD965_RS02885	-	Spore coat protein F-like protein YraD	M
JD965_RS02890	-	uncharacterized protein	S
JD965_RS02895	1.1.1.-	Uncharacterized zinc-type alcohol dehydrogenase-like protein AdhB	E
JD965_RS02900	-	Spore coat protein F-like protein YraF	M
JD965_RS02905	-	Spore coat protein F-like protein YraG	S
JD965_RS04370	-	Spore coat protein	O
JD965_RS04950	-	Spore coat protein F-like protein YgzC	M
JD965_RS04950	-	Spore coat protein F-like protein YgzC	M
JD965_RS05185	-	Spore coat protein F-like protein YhcQ	M
JD965_RS05185	-	Spore coat protein F-like protein YhcQ	M
JD965_RS05465	-	Endospore coat-associated protein YheC	S
JD965_RS06370	-	Spore coat protein	M
JD965_RS06375	-	Spore coat protein	
JD965_RS06380	-	Spore coat protein Y	
JD965_RS06385	-	Spore coat protein X	S
JD965_RS06390	-	Spore coat protein	
JD965_RS06395	-	Spore coat protein	S
JD965_RS06400	-	Sporulation protein YjcA	S
JD965_RS09110	-	Spore coat protein	M
JD965_RS09565	-	Spore coat protein	O
JD965_RS10920	-	Spore coat protein	S
JD965_RS12190	-	Spore coat-associated protein	S
JD965_RS13235	-	Spore coat assembly protein ExsA	S
JD965_RS15730	-	Endospore coat-associated protein YutH	S
JD965_RS17670	-	Spore coat protein	S
JD965_RS17675	-	Inner spore coat protein	M
JD965_RS17680	-	Spore coat protein	S
JD965_RS19680	-	Spore coat protein	M
Spore Wall			
JD965_RS18480	-	Spore coat polysaccharide biosynthesis protein SpsG	M
JD965_RS18485	-	Spore coat polysaccharide biosynthesis protein SpsF	M
JD965_RS18505	-	Spore coat polysaccharide biosynthesis protein SpsB	M
Spore germination			
JD965_RS00120	-	Spore germination protein YaaH	M
JD965_RS00935	-	Spore germination protein GerD	S
JD965_RS01855	-	Spore germination protein KA	S
JD965_RS01860	-	Spore germination protein KC	S
JD965_RS01865	-	Spore germination protein KB	S

Gene locus	EC. No.	Product	COG
JD965_RS02095	-	Spore germination lipase LipC	E
JD965_RS05910	-	Probable spore germination protein GerPF	S
JD965_RS05915	-	Probable spore germination protein GerPE	S
JD965_RS05920	-	Probable spore germination protein GerPD	S
JD965_RS05925	-	Probable spore germination protein GerPC	S
JD965_RS05930	-	Probable spore germination protein GerPB	S
JD965_RS05935	-	Probable spore germination protein GerPA	S
JD965_RS10290	-	Spore germination protein GerT	O
JD965_RS11980	-	Spore germination protein-like protein YpzD	
JD965_RS11985	-	Spore germination protein-like protein YpzD	
JD965_RS13785	-	Spore germination protein GerM	S
JD965_RS13800	-	Spore germination protein GerE	K
JD965_RS15490	-	Spore germination protein-like protein YueG	S
JD965_RS16165	-	Spore germination protein A1	S
JD965_RS16170	-	Spore germination protein A2	S
JD965_RS16175	-	Spore germination protein A3	S
JD965_RS17535	-	Spore germination protein	S
JD965_RS17540	-	Spore germination protein B2	S
JD965_RS17545	-	Spore germination protein B3	S
JD965_RS18635	-	Spore morphoproteinis and germination protein YwcE	
Dipicolinic acid			
JD965_RS18515	-	Spore coat protein GerQ	S
Spore others			
JD965_RS00370	-	Spore protein YabP	S
JD965_RS00375	-	Spore protein YabQ	S
JD965_RS04575	-	Small, acid-soluble spore protein	M
JD965_RS04765	-	Small, acid-soluble spore protein gamma-type	S
JD965_RS05450	-	Small, acid-soluble spore protein	S
JD965_RS07320	-	Small, acid-soluble spore protein	S
JD965_RS07550	4.1.99.14	Spore photoproduct lyase	L
JD965_RS09570	-	Small, acid-soluble spore protein	M
JD965_RS09575	-	Small, acid-soluble spore protein	M
JD965_RS09595	-	Small, acid-soluble spore protein	
JD965_RS09600	-	Small, acid-soluble spore protein Tlp	M
JD965_RS10800	-	Small, acid-soluble spore protein	
JD965_RS10970	-	Small, acid-soluble spore protein	
JD965_RS11405	-	Spore maturation protein	S
JD965_RS11410	-	Spore maturation protein	S
JD965_RS13925	-	Small, acid-soluble spore protein	M
JD965_RS14300	-	Uncharacterized spore protein YtfJ	S

Gene locus	EC. No.	Product	COG
JD965_RS14335	-	Small, acid-soluble spore protein	S
JD965_RS16310	-	Small, acid-soluble spore protein J	
JD965_RS18345	-	Prespore-specific transcriptional regulator RsfA	K
Health enhancement			
JD965_RS07825	-	Putative gamma-glutamylcyclotransferase YkqA	S
JD965_RS02700	1.14.99.5<br>0	Gamma-glutamyl hercynylcysteine S-oxide synthase	S
JD965_RS06080	1.2.1.38	N-acetyl-gamma-glutamyl-phosphate reductase	E
JD965_RS01675	1.2.1.88	L-glutamate gamma-semialdehyde dehydrogenase	C
JD965_RS18425	1.2.1.88	L-glutamate gamma-semialdehyde dehydrogenase	C
JD965_RS03675	2.3.2.13	Protein-glutamine gamma-glutamyltransferase	E
JD965_RS15205	2.3.2.13	Protein-glutamine gamma-glutamyltransferase	S
JD965_RS09945	2.3.2.2,<br>3.4.19.13	Gamma-glutamyltransferase	E
JD965_RS17695	2.3.2.2,<br>3.4.19.13	Gamma-glutamyltransferase	E
JD965_RS06465	2.5.1.48	Cystathionine gamma-synthase	E
JD965_RS01465	3.1.1.3	Triacylglycerol lipase	S
JD965_RS14670	3.2.1.22	Alpha-galactosidase	G
JD965_RS10245	3.4.-.-	D-gamma-glutamyl-meso-diaminopimelic acid endopeptidase CwlS	M|M
JD965_RS17575	3.4.-.-	Gamma-DL-glutamyl hydrolase	M
JD965_RS15585	3.4.11.1	Leucyl aminopeptidase	E
JD965_RS07110	3.4.14.13	Gamma-D-glutamyl-L-lysine dipeptidyl-peptidase	M
JD965_RS12300	3.4.19.11	Gamma-D-glutamyl-meso-diaminopimelate peptidase	E
JD965_RS05730	3.4.21.62	Subtilisin	O
JD965_RS00205	4.1.1.19	Arginine decarboxylase	E
JD965_RS07910	4.1.1.19	Arginine decarboxylase	E
JD965_RS09315	4.4.1.1	Cystathionine gamma-lyase	P
JD965_RS12945	4.4.1.1,<br>4.4.1.2	Cystathionine gamma-lyase	E

The Enzyme Commission (EC) number is a numerical classification scheme for enzymes, based on the chemical reactions they catalyze. The Clusters of Orthologus Group (COG) categorization was generated by annotated gene functions.

Table S3. List of the genes for amino acid synthesis.

Gene locus	EC No.	Product	Gene	COG
Amino acid synthesis				
JD965_RS00440	5.2.1.8	Peptidylprolyl isomerase	*prsA*	O
JD965_RS00445	2.5.1.47	Cysteine synthase	*cysK*	E
JD965_RS00460	2.6.1.42	Branched-chain-amino-acid transaminase	*ilvE*	E
JD965_RS00620	2.3.1.30	Serine O-acetyltransferase	*cysE*	E
JD965_RS01085	2.5.1.47	Cysteine synthase	*cysK*	K
JD965_RS01345	2.6.1.42	Branched-chain-amino-acid transaminase	*ilvE*	E
JD965_RS01355	2.1.1.10	Homocysteine S-methyltransferase	*mmuM*	E
JD965_RS01365	3.5.1.2	Glutaminase	*glsA*	E
JD965_RS01655	2.7.1.71	Shikimate kinase	*aroKL*	E
JD965_RS01940	2.7.2.4	Aspartate kinase	*lysC*	E
JD965_RS02000	6.3.5.4	Asparagine synthase (glutamine-hydrolyzing)	*asnB*	E
JD965_RS03700	2.6.1.1	Aspartate transaminase	*aspB*	E
JD965_RS04315	1.14.13.1 65	Nitric-oxide synthase (NAD(P)H)	*nos*	EP
JD965_RS04415	1.1.1.25	Shikimate dehydrogenase	*aroE*	E
JD965_RS04420	4.2.1.10	3-dehydroquinate dehydratase	*aroD*	E
JD965_RS05365	2.6.1.1	Aspartate transaminase	*yhdR*	E
JD965_RS05415	2.6.1.21	D-amino-acid transaminase	*dat*	E
JD965_RS05555	5.2.1.8	Peptidylprolyl isomerase	*prsA*	O
JD965_RS05960	6.3.5.4	Asparagine synthase (glutamine-hydrolyzing)	*asnB*	E
JD965_RS06005	2.1.1.10	Homocysteine S-methyltransferase	*mmuM*	E
JD965_RS06110	2.1.3.3	Ornithine carbamoyltransferase	*argFI*	E
JD965_RS06465	2.5.1.48	Cystathionine gamma-synthase	*metB*	E
JD965_RS06470	4.4.1.13	Cystathionine beta-lyase	*metC*	E
JD965_RS07155	2.7.2.11	Glutamate 5-kinase	*proB*	E
JD965_RS07160	1.2.1.41	Glutamate-5-semialdehyde dehydrogenase	*proA*	E
JD965_RS07215	2.1.1.14	5-methyltetrahydropteroyltriglutamate--homocysteine S-methyltransferase	*metE*	E
JD965_RS07215	2.1.1.14	5-methyltetrahydropteroyltriglutamate--homocysteine S-methyltransferase	*metE*	E
JD965_RS07585	2.6.1.-	Putative N-acetyl-LL-diaminopimelate aminotransferase	*patA*	E
JD965_RS07675	2.3.1.89	Tetrahydrodipicolinate N-acetyltransferase	*dapHD*	E
JD965_RS07680	3.5.1.47	N-acetyldiaminopimelate deacetylase	*dapL*	E
JD965_RS07770	2.7.1.11	6-phosphofructokinase	*pfkB*	G
JD965_RS07930	3.1.3.253 .1.3.933. 1.3.15	Inositol-phosphate phosphatase	IMPL2	S
JD965_RS08010	3.5.1.2	Glutaminase	*glsA*	E
JD965_RS08025	6.4.1.1	Pyruvate carboxylase	*pyc*	C
JD965_RS08965	1.2.1.11	Aspartate-semialdehyde dehydrogenase	*asd*	E

Gene locus	EC No.	Product	Gene	COG
JD965_RS08970	2.7.2.4	Aspartate kinase	*lysC*	E
JD965_RS08975	4.3.3.7	4-hydroxy-tetrahydrodipicolinate synthase	*dapA*	E
JD965_RS09315	4.4.1.1	Cystathionine gamma-lyase	CTH	P
JD965_RS09325	6.3.1.2	Glutamate--ammonia ligase	*glnA*	E
JD965_RS09525	2.2.1.1	Transketolase	*tktAB*	G
JD965_RS09940	5.3.1.9	Glucose-6-phosphate isomerase	*pgi*	S
JD965_RS09975	1.4.1.131<br>.4.1.14	Glutamate synthase (NADPH)	*gltD*	E
JD965_RS09980	1.4.1.131<br>.4.1.14	Glutamate synthase (NADPH)	*gltB*	E
JD965_RS09990	2.7.2.11	Glutamate 5-kinase	*proB*	E
JD965_RS09995	1.5.1.2	Pyrroline-5-carboxylate reductase	*proC*	E
JD965_RS10680	4.3.1.19	Threonine ammonia-lyase	*ilvA*	E
JD965_RS10725	4.2.1.9	Dihydroxy-acid dehydratase	*ilvD*	E
JD965_RS10745	2.3.1.46	Homoserine O-succinyltransferase	*metA*	E
JD965_RS10940	2.7.1.69	Protein-N(pi)-phosphohistidine--sugar phosphotransferase	*crr*	G
JD965_RS11010	2.6.1.1	Aspartate transaminase	*aspB*	E
JD965_RS11070	1.17.1.8	4-hydroxy-tetrahydrodipicolinate reductase	*dapB*	E
JD965_RS11130	2.5.1.19	3-phosphoshikimate 1-carboxyvinyltransferase	*aroA*	E
JD965_RS11140	2.6.1.9	Histidinol-phosphate transaminase	*hisC*	E
JD965_RS11145	4.2.1.20	Tryptophan synthase	*trpA*	E
JD965_RS11150	4.2.1.20	Tryptophan synthase	*trpB*	E
JD965_RS11155	5.3.1.24	Phosphoribosylanthranilate isomerase	*trpF*	E
JD965_RS11160	4.1.1.48	Indole-3-glycerol-phosphate synthase	*trpC*	E
JD965_RS11165	2.4.2.18	Anthranilate phosphoribosyltransferase	*trpD*	E
JD965_RS11170	4.1.3.27	Anthranilate synthase	*trpE*	E
JD965_RS11180	4.2.3.4	3-dehydroquinate synthase	*aroB*	E
JD965_RS11185	4.2.3.5	Chorismate synthase	*aroC*	E
JD965_RS11495	4.1.1.20	Diaminopimelate decarboxylase	*lysA*	E
JD965_RS11695	5.4.2.12	Phosphoglycerate mutase (2,3-diphosphoglycerate-independent)	*gpmB*	E
JD965_RS11720	1.5.1.2	Pyrroline-5-carboxylate reductase	*proC*	E
JD965_RS11800	4.3.1.3	Histidine ammonia-lyase	*hutH*	E
JD965_RS12715	1.1.1.25	Shikimate dehydrogenase	*aroE*	E
JD965_RS12945	4.4.1.2	Cystathionine gamma-lyase	*mccB*	E
JD965_RS12950	2.5.1.47	Cysteine synthase	*cysK*	E
JD965_RS13265	5.4.99.54<br>.2.1.51	Chorismate mutase	*pheA*	E
JD965_RS13270	5.4.99.5	Chorismate mutase	*pheB*	E
JD965_RS13695	4.2.1.334<br>.2.1.35	3-isopropylmalate dehydratase	*leuD*	E
JD965_RS13700	4.2.1.334<br>.2.1.35	3-isopropylmalate dehydratase	*leuC*	E

Gene locus	EC No.	Product	Gene	COG
JD965_RS13705	1.1.1.85	3-isopropylmalate dehydrogenase	*leuB*	E
JD965_RS13715	1.1.1.86	Ketol-acid reductoisomerase (NADP(+))	*ilvC*	E
JD965_RS13720	2.2.1.6	acetolactate synthase small subunit	*ilvN*	E
JD965_RS13725	2.2.1.6	Acetolactate synthase large subunit	*IlvB*	E
JD965_RS13830	2.7.2.4	Aspartate kinase	*lysC*	E
JD965_RS14090	1.2.1.12	Glyceraldehyde-3-phosphate dehydrogenase (phosphorylating)	*gapA*	G
JD965_RS14195	2.7.1.40	Pyruvate kinase	*pyk*	G
JD965_RS14200	2.7.1.11	6-phosphofructokinase	*pfkA*	G
JD965_RS14265	4.3.2.1	Argininosuccinate lyase	*argH*	E
JD965_RS14270	6.3.4.5	Argininosuccinate synthase	*argG*	E
JD965_RS14495	5.4.99.54 .2.1.51	Chorismate mutase	*pheA*	E
JD965_RS14815	2.5.1.47	Cysteine synthase	*cysK*	E
JD965_RS15265	5.3.1.9	Glucose-6-phosphate isomerase	*pgi*	G
JD965_RS15320	4.4.1.13	Cystathionine beta-lyase	*patB*	E
JD965_RS15680	5.1.1.7	Diaminopimelate epimerase	*dapF*	E
JD965_RS15715	2.7.1.39	Homoserine kinase	*thrB1*	E
JD965_RS15720	4.2.3.1	Threonine synthase	*thrC*	E
JD965_RS15725	1.1.1.3	Homoserine dehydrogenase	*hom*	E
JD965_RS16060	1.5.-.-	Proline dehydrogenase	*putB*	E
JD965_RS16665	4.2.1.11	Phosphopyruvate hydratase	*eno*	G
JD965_RS16670	5.4.2.12	Phosphoglycerate mutase (2,3-diphosphoglycerate-independent)	*gpmI*	G
JD965_RS16680	2.7.2.3	Phosphoglycerate kinase	*pgk*	G
JD965_RS16685	1.2.1.12	Glyceraldehyde-3-phosphate dehydrogenase (phosphorylating)	*gapA*	G
JD965_RS17085	3.6.1.313 .5.4.19	Phosphoribosyl-ATP diphosphatase	*hisIE*	E
JD965_RS17090	4.1.3.-	Imidazole glycerol phosphate synthase subunit HisF	*hisF*	E
JD965_RS17095	5.3.1.16	1-(5-phosphoribosyl)-5-((5-phosphoribosylamino)methylideneamino)imidazole-4-carboxamideisomerase	*hisA*	E
JD965_RS17100	2.4.2.-	Imidazole glycerol phosphate synthase subunit HisH	*hisH*	E
JD965_RS17105	4.2.1.19	Imidazoleglycerol-phosphate dehydratase	*hisB*	E
JD965_RS17110	1.1.1.23	Histidinol dehydrogenase	*hisD*	E
JD965_RS17115	2.4.2.17	ATP phosphoribosyltransferase	*hisG*	E
JD965_RS17120	-	ATP phosphoribosyltransferase regulatory subunit	*hisZ*	E
JD965_RS18035	2.1.2.1	Glycine hydroxymethyltransferase	*glyA*	E
JD965_RS18145	4.1.2.13	Fructose-bisphosphate aldolase	*fbaA*	G
JD965_RS18805	2.6.1.42	Branched-chain-amino-acid transaminase	*ilvE*	E
JD965_RS18955	2.1.1.14	5-methyltetrahydropteroyltriglutamate--homocysteine S-methyltransferase	*metE*	E
JD965_RS19485	3.5.3.1	Arginase	*rocF*	E
JD965_RS19495	2.6.1.13	Ornithine aminotransferase	*rocD*	E

Gene locus	EC No.	Product	Gene	COG
GABA				
JD965_RS00205	4.1.1.19	Arginine decarboxylase	*speA*	
JD965_RS02925	3.5.1.94	gamma-glutamyl-gamma-aminobutyrate hydrolase family protein	*puuD*	S
JD965_RS04105	1.2.1.-	Gamma-glutamyl-aminobutyraldehyde dehydrogenase	*puuC*	C
JD965_RS05765	1.4.3.-	Gamma-glutamyl-putrescine oxidase	*puuB*	CE
JD965_RS07910	4.1.1.19	Arginine decarboxylase	*speA*	
JD965_RS09325	6.3.1.11	Gamma-glutamyl-putrescine synthetase	*puuA*	E
JD965_RS10190	1.2.1.-	Gamma-glutamyl-aminobutyraldehyde dehydrogenase	*puuC*	C
JD965_RS18285	3.5.3.11	Agmatinase	*speB*	
JD965_RS19330	1.2.1.-	Gamma-glutamyl-aminobutyraldehyde dehydrogenase	*puuC*	C
Branched chain fatty acid				
JD965_RS00460	2.6.1.42	Branched-chain-amino-acid transaminase	*ilvE*	E
JD965_RS01345	2.6.1.42	Branched-chain-amino-acid transaminase	*ilvE*	E
JD965_RS11870	2.3.1.168	Dihydrolipoyllysine-residue (2-methylpropanoyl)transferase	*bkdB*	C
JD965_RS11875	1.2.4.4	3-methyl-2-oxobutanoate dehydrogenase (2-methylpropanoyl-transferring)	*bkdA2*	C
JD965_RS11880	1.2.4.4	3-methyl-2-oxobutanoate dehydrogenase (2-methylpropanoyl-transferring)	*bkdA1*	C
JD965_RS11895	1.4.1.9	Leucine dehydrogenase	*leu*	E

Table S4. List of the genes for whitening and scavenging of reactive oxygen species.

Gene locus	EC No.	Product	Gene	COG
Uracil				
JD965_RS00325	2.7.6.1	Ribose-phosphate diphosphokinase	*prsA*	F
JD965_RS01365	3.5.1.2	Glutaminase	*glsA*	E
JD965_RS02980	2.3.1.12	Dihydrolipoyllysine-residue acetyltransferase	*pdhC*	S
JD965_RS03540	1.1.1.49<br>1.1.1.363	Glucose-6-phosphate dehydrogenase (NADP(+))	*zwf*	G
JD965_RS03690	6.3.5.5	Carbamoyl-phosphate synthase (glutamine-hydrolyzing)	*carB*	S
JD965_RS03830	1.4.1.13	Glutamate synthase (NADPH)	*gltB*	E
JD965_RS04560	2.3.1.61	Dihydrolipoyllysine-residue succinyltransferase	*sucB*	C
JD965_RS04565	1.8.1.4	Dihydrolipoyl dehydrogenase	*pdhD*	C
JD965_RS05315	2.3.3.1	Citrate (Si)-synthase	*gltA*	C
JD965_RS06100	6.3.5.5	Carbamoyl-phosphate synthase (glutamine-hydrolyzing)	*carA*	F
JD965_RS06105	6.3.5.5	Carbamoyl-phosphate synthase (glutamine-hydrolyzing)	*carB*	F
JD965_RS07120	3.1.1.31	6-phosphogluconolactonase	*pgl*	G
JD965_RS07770	2.7.1.11	6-phosphofructokinase	*pfkB*	G
JD965_RS07875	1.2.4.1	Pyruvate dehydrogenase (acetyl-transferring)	*pdhA*	C
JD965_RS07880	1.2.4.1	Pyruvate dehydrogenase (acetyl-transferring)	*pdhB*	C
JD965_RS07885	2.3.1.12	Dihydrolipoyllysine-residue acetyltransferase	*pdhC*	C
JD965_RS07890	1.8.1.4	Dihydrolipoyl dehydrogenase	*pdhD*	C
JD965_RS08010	3.5.1.2	Glutaminase	*glsA*	E
JD965_RS08320	2.4.2.9	Uracil phosphoribosyltransferase	*pyrR*	F
JD965_RS08330	2.1.3.2	Aspartate carbamoyltransferase	*pyrB*	F
JD965_RS08335	3.5.2.3	Dihydroorotase	*pyrC*	F
JD965_RS08340	6.3.5.5	Carbamoyl-phosphate synthase (glutamine-hydrolyzing)	*carA*	F
JD965_RS08345	6.3.5.5	Carbamoyl-phosphate synthase (glutamine-hydrolyzing)	*carB*	F
JD965_RS08350	-	Dihydroorotate dehydrogenase B (NAD(+)), electron transfer subunit	*pyrDI I*	C
JD965_RS08360	4.1.1.23	Orotidine-5'-phosphate decarboxylase	*pyrF*	F
JD965_RS08365	2.4.2.10	Orotate phosphoribosyltransferase	*pyrE*	F
JD965_RS08630	6.2.1.5	Succinate--CoA ligase (ADP-forming)	*sucC*	C
JD965_RS09325	6.3.1.2	Glutamine synthetase	*glnA*	E
JD965_RS09580	4.2.1.3	Aconitate hydratase	*acnA*	C
JD965_RS09940	5.3.1.9	Glucose-6-phosphate isomerase	*pgi*	S
JD965_RS09975	1.4.1.13	Glutamate synthase (NADPH)	*gltD*	E
JD965_RS09980	1.4.1.13	Glutamate synthase (NADPH)	*gltB*	E|E|E
JD965_RS10220	2.3.1.61	Dihydrolipoyllysine-residue succinyltransferase	*sucB*	C
JD965_RS10225	1.2.4.2	Oxoglutarate dehydrogenase (succinyl-transferring)	*sucA*	C
JD965_RS11750	1.1.1.44<br>1.1.1.343	Phosphogluconate dehydrogenase (NADP(+)-dependent, decarboxylating)	*gnd*	G
JD965_RS11885	1.8.1.4	Dihydrolipoyl dehydrogenase	*pdhD*	C

Gene locus	EC No.	Product	Gene	COG
JD965_RS11925	2.3.3.1	Citrate (Si)-synthase	*gltA*	C
JD965_RS12305	2.7.1.2	Glucokinase	*glk*	G
JD965_RS13810	1.3.5.1<br>1.3.5.4	Succinate dehydrogenase (quinone)	*frdB*	C
JD965_RS13815	1.3.5.1<br>1.3.5.4	Succinate dehydrogenase (quinone)	*frdA*	C
JD965_RS13820	-	Succinate dehydrogenase cytochrome b558 subunit	*frdC*	C
JD965_RS14090	1.2.1.12	Glyceraldehyde-3-phosphate dehydrogenase (phosphorylating)	*gapA*	G
JD965_RS14140	1.1.1.37	Malate dehydrogenase	*mdh*	C
JD965_RS14145	1.1.1.42	Isocitrate dehydrogenase (NADP(+))	*icd*	C
JD965_RS14150	2.3.3.1	Citrate (Si)-synthase	*gltA*	C
JD965_RS14195	2.7.1.40	Pyruvate kinase	*pyk*	G
JD965_RS14200	2.7.1.11	6-phosphofructokinase	*pfkA*	G
JD965_RS15265	5.3.1.9	Glucose-6-phosphate isomerase	*pgi*	G
JD965_RS15790	3.5.2.3	Dihydroorotase	*pyrC*	F
JD965_RS16155	4.2.1.2	Fumarate hydratase	*fumC*	C
JD965_RS16665	4.2.1.11	Phosphopyruvate hydratase	*eno*	G
JD965_RS16670	-	Phosphoglycerate mutase (2,3-diphosphoglycerate-independent)	*gpmI*	G
JD965_RS16675	5.3.1.1	Triose-phosphate isomerase	*tpiA*	G
JD965_RS16680	2.7.2.3	Phosphoglycerate kinase	*pgk*	G
JD965_RS16685	1.2.1.12	Glyceraldehyde-3-phosphate dehydrogenase (phosphorylating)	*gapA*	G
JD965_RS17515	6.3.5.5	Carbamoyl-phosphate synthase (glutamine-hydrolyzing)	*carB*	F
JD965_RS18030	2.4.2.9	Uracil phosphoribosyltransferase	*upp*	F
JD965_RS18045	3.5.1.6	Ribose-5-phosphate isomerase	*rpiB*	G
JD965_RS18145	4.1.2.13	Fructose-bisphosphate aldolase	*fbaA*	G
Lipoteichoic acid				
JD965_RS17400	2.7.8.33<br>2.7.8.35	UDP-N-acetylglucosamine--undecaprenyl-phosphateN-acetylglucosaminephosphotransferase	*tagO*	M
JD965_RS17405	2.4.-.-	Putative teichuronic acid biosynthesis glycosyltransferase TuaH	*tuaH*	M
JD965_RS17410	2.4.-.-	Putative teichuronic acid biosynthesis glycosyltransferase TuaG	*tuaG*	M
JD965_RS17415		Teichuronic acid biosynthesis protein TuaF		
JD965_RS17420		Teichuronic acid biosynthesis protein TuaE		S
JD965_RS17425	1.1.1.22	UDP-glucose 6-dehydrogenase	*ugd*	M
JD965_RS17430	2.4.-.-	Putative teichuronic acid biosynthesis glycosyltransferase TuaC	*tuaC*	M
JD965_RS17435		Teichuronic acid biosynthesis protein TuaB		M
JD965_RS17440		Putative undecaprenyl-phosphate N-acetylgalactosaminyl 1-phosphate transferase		M
JD965_RS17445		Transcriptional regulator LytR		K
JD965_RS17450	5.1.3.14	UDP-N-acetylglucosamine 2-epimerase (non-hydrolyzing)	*wecB*	M
JD965_RS17455	2.7.7.9	UTP--glucose-1-phosphate uridylyltransferase	*galF*	M

Gene locus	EC No.	Product	Gene	COG
JD965_RS17460	3.6.3.40	Teichoic-acid-transporting ATPase	*tagH*	GM
JD965_RS17465		Teichoic acid translocation permease protein TagG		V
JD965_RS17470	2.7.8.12	Teichoic acid poly(glycerol phosphate) polymerase	*tagF*	M|M
JD965_RS17475		hypothetical protein		
JD965_RS17480	2.7.8.12	Teichoic acid poly(glycerol phosphate) polymerase	*tagF*	M
JD965_RS17485	2.7.7.39	Glycerol-3-phosphate cytidylyltransferase	*tagD*	IM
JD965_RS17490	2.4.1.187	N-acetylglucosaminyldiphosphoundecaprenol N-acetyl-beta-D-mannosaminyltransferase	*tagA*	M
JD965_RS17495	2.7.8.44	Teichoic acid glycerol-phosphate primase	*tagB*	M
JD965_RS17500	3.2.1.96	Mannosyl-glycoprotein endo-beta-N-acetylglucosaminidase		G
JD965_RS17505	2.4.-.-	Putative glycosyltransferase EpsE	*exoO*	M
JD965_RS17510	2.6.1.33	dTDP-4-amino-4,6-dideoxy-D-glucose transaminase	*vioA*	E
JD965_RS17515	6.3.5.5	Carbamoyl-phosphate synthase (glutamine-hydrolyzing)	*carB*	F
JD965_RS17520	2.7.8.12	Teichoic acid poly(glycerol phosphate) polymerase	*tagF*	M
JD965_RS18780		Uncharacterized membrane protein YwzH		
JD965_RS18785	6.1.1.13	D-alanine--poly(phosphoribitol) ligase	*dltA*	Q
JD965_RS18790		Protein DltB		M
JD965_RS18795	6.1.1.13	D-alanine--poly(phosphoribitol) ligase	*dltC*	I
JD965_RS18800		Protein DltD	*dltD*	M
Glutathione				
JD965_RS04955	3.4.11.2	Membrane alanyl aminopeptidase	*pepN*	E
JD965_RS05000	1.11.1.6	Catalase	*kat*	P
JD965_RS09945	2.3.2.2<br>3.4.19.13	Gamma-glutamyltransferase	*ggt*	E
JD965_RS10200	1.15.1.1	Superoxide dismutase	*sod*	P
JD965_RS10240	1.15.1.1	Superoxide dismutase	*sod*	P
JD965_RS10740	1.11.1.9	Glutathione peroxidase	*gpx*	O
JD965_RS12385	1.15.1.1	Superoxide dismutase	*sod*	P
JD965_RS15585	3.4.11.1	Leucyl aminopeptidase	*pepA*	E
JD965_RS17695	2.3.2.2<br>3.4.19.13	Gamma-glutamyltransferase	*ggt*	E
JD965_RS18865	1.11.1.6	Catalase	*kat*	P
JD965_RS18980	1.11.1.6	Catalase	*kat*	P
Glycogen synthase kinase-3 gene				
JD965_RS00410	2.7.11.1	Non-specific serine/threonine protein kinase		T
JD965_RS01195	2.7.11.1	Non-specific serine/threonine protein kinase		T
JD965_RS02425	2.7.11.1	Non-specific serine/threonine protein kinase		T
JD965_RS02440	2.7.11.1	Non-specific serine/threonine protein kinase		T
JD965_RS07570	2.7.11.1	Non-specific serine/threonine protein kinase		S
JD965_RS08470	2.7.11.1	Non-specific serine/threonine protein kinase		T|T
JD965_RS11540	2.7.11.1	Non-specific serine/threonine protein kinase		T

Gene locus	EC No.	Product	Gene	COG
JD965_RS19335	2.7.11.1	Non-specific serine/threonine protein kinase		S
